# Supplementary material for: Assessing the impact of rising child poverty on the unprecedented rise in infant mortality in England, 2000–2017: time trend analysis
Source: BMJ Open. 2019 Oct 2;9(10):e029424. doi: 10.1136/bmjopen-2019-029424 (PMC6954495; doi:10.1136/bmjopen-2019-029424)
Supplement: Supplementary data [file bmjopen-2019-029424supp001.pdf]

Supplementary Material: Child poverty and inequalities in infant mortality in England 2000-17: time trend analysis

Web appendix 1: Summary of dataset for local authority level analysis

Statistic	N	Mean	St. Dev.	Min	Max
Year	5,832	2,008	5	2,000	2,017
birth	5,832	1,975	1,605	222	17,766
death	5,832	9	11	0	169
imd_quintile	5,832	3	1	1	5
year_centred	5,832	8	5	0	17
year_knot13	5,832	1	1	0	4
imr	5,832	418	214	0	1,570

Web appendix 2: Jointpoint regression to identify breakpoint

We therefore investigated empirically whether there was a significant change in the trend in infant mortality between 2000 and 2017. We use an iterative search procedure to identify which breakpoint produced the best fitting mode by comparing all models with these alternative breakpoints at each year from 2001 to 2016, as well as models no breakpoints. We then plotted the Residual Mean Squared Error (RMSE) values from each of these models to identify the model that provided the best fit with the data. In other words we fitted 16 separate models each with a different break points. The figure below shows the RMSE from each of these models – indicating that a breakpoint at 2013 provides the best fitting model compared to all the other alternative break points.

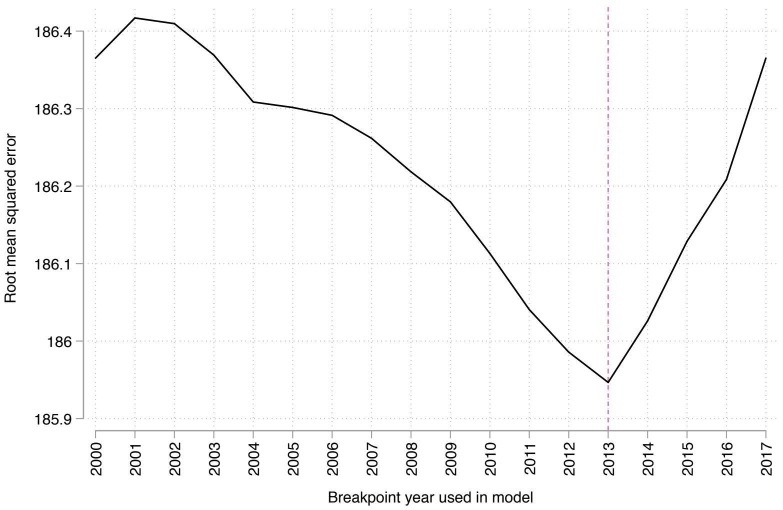

Taylor-Robinson D, *et al. BMJ Open* 2019; 9:e029424. doi: 10.1136/bmjopen-2019-029424

Web appendix 3: Segmented mixed effects regression model for infant mortality including two linear spline terms

We use a segmented mixed effects regression model for the mean response:

Y_{ij} = β$_1$* spline1+ β$_2$*spline2 + β$_2$*spline1*IMDQ+ β$_4$*spline2*IMDQ + U_i + Z_{ij}

Let Y_{ij} is the *j*th repeated measurement (here, IMR) on the *i*th local area. Spline1 takes the value of year. Spline2 takes the value of *0* for year less than *2013*, taken to be the breakpoint for change in tren*d, and year − 2013* for year greater than *2013. This* defines a segmented regression with a change in slope of IMR at year=2013. IMDQ is IMD quintile, coded as a categorical variable. U_i is a random intercept term describing how the average IMR of the *i*th local area varies about the population-averaged response for all local areas with the same values of the explanatory variables. The model assumes that the U_i are independent copies of a Normally distributed random variable with mean zero and variance v^2. Z_{ij} is an error term that assumes that the Z_{ij} are independent copies of a Normally distributed random variable with mean zero and variance τ^2.

Web appendix 4: Link showing show the distribution of infant mortality across England
https://dblalex.carto.com/builder/c81de0d5-f893-4109-9471-b4b55afa351e/embed

Web appendix 5: Linear mixed effects model results for trends in infant mortality at lower tier local authority level 2000-2017 – full R output with random effects.

	imr		
Predictors	*Estimates*	*CI*	*p*
(Intercept)	377.45	349.94 – 404.96	**<0.001**
year centred	-5.84	-8.70 – -2.98	**<0.001**
year knot 13	-4.19	-16.92 – 8.54	0.519
factor(imd quintile)2	66.13	27.38 – 104.89	**0.001**
factor(imd quintile)3	109.87	71.12 – 148.62	**<0.001**
factor(imd quintile)4	170.67	131.91 – 209.42	**<0.001**
factor(imd quintile)5	292.08	253.33 – 330.83	**<0.001**
year_centred:factor(imd_quintile)2	-2.67	-6.70 – 1.36	0.194
year_centred:factor(imd_quintile)3	-2.56	-6.59 – 1.47	0.213
year_centred:factor(imd_quintile)4	-7.27	-11.30 – -3.24	**<0.001**
year_centred:factor(imd_quintile)5	-12.43	-16.46 – -8.40	**<0.001**
year_knot13:factor(imd_quintile)2	15.65	-2.28 – 33.58	0.087
year_knot13:factor(imd_quintile)3	3.89	-14.04 – 21.83	0.671
year_knot13:factor(imd_quintile)4	20.50	2.57 – 38.43	**0.025**
year_knot13:factor(imd_quintile)5	24.14	6.20 – 42.07	**0.008**
**Random Effects**			
σ^2	34654.53		
$\tau_{00\ LA\_name}$	3953.12		

ICC $_{LA\_name}$	0.10
Observations	5832
Marginal R^2 / Conditional R^2	0.159 / 0.245

Web appendix 6: Residual diagnostics for local authority level analysis

The residuals from our longitudinal model plotted below. The residuals are normally distributed.

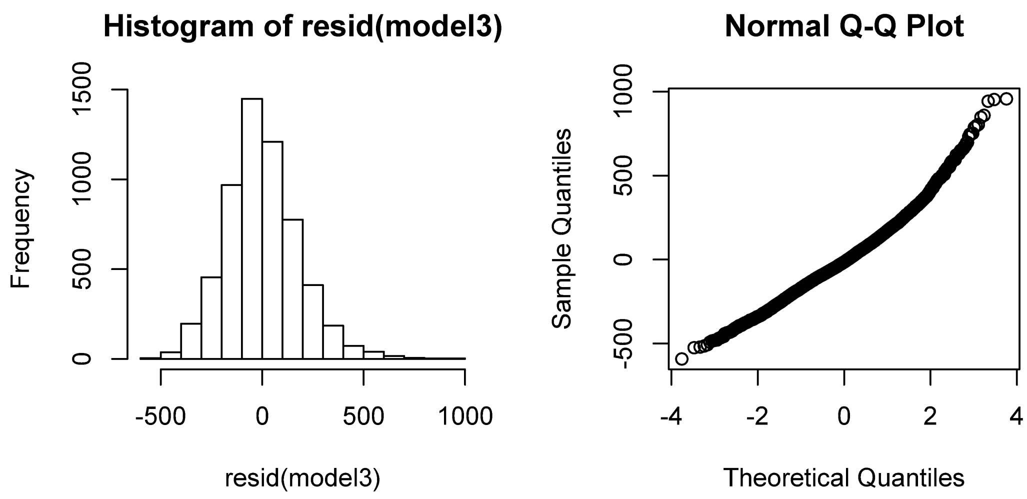

Web appendix 7: Modelling strategy for the fixed effects level analysis

Our cross-regional fixed effects model is as follows.

$Y_{ij} = \beta_1 \text{Child Poverty}_{ij} + \boldsymbol{\beta}_2 \text{Year} + \mu_i + Z_{ij}$

Let Y_{ij} is the infant mortality rate in the jth year in the ith English region,

Child Poverty$_{ij}$ is the child poverty rate in the jth year in the ith English region.

Year is a set of dummy variables for each year from 2000 to 2017

μ are regional fixed effects, and Z_{ij} are independent copies of a normally distributed random error term.

Web appendix 8: Residual diagnostics for regional fixed effects analysis

Taylor-Robinson D, *et al. BMJ Open* 2019; 9:e029424. doi: 10.1136/bmjopen-2019-029424

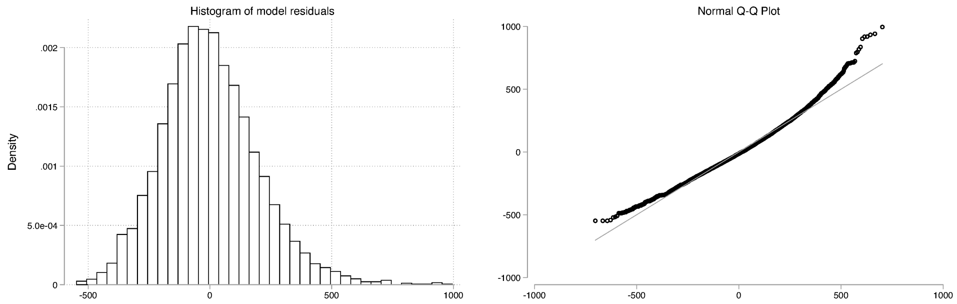

Web appendix 9: Estimates from table 1 plotted with their confidence intervals

Plotting the estimates for annual change (deaths per 100,000) in infant mortality 2014-2017 relative to previous trend (2000-2013) and their confidence intervals by small area deprivation quintile (most deprived=quintile 5) suggests that the data are compatible with a monotonic dose response relationship with deprivation.

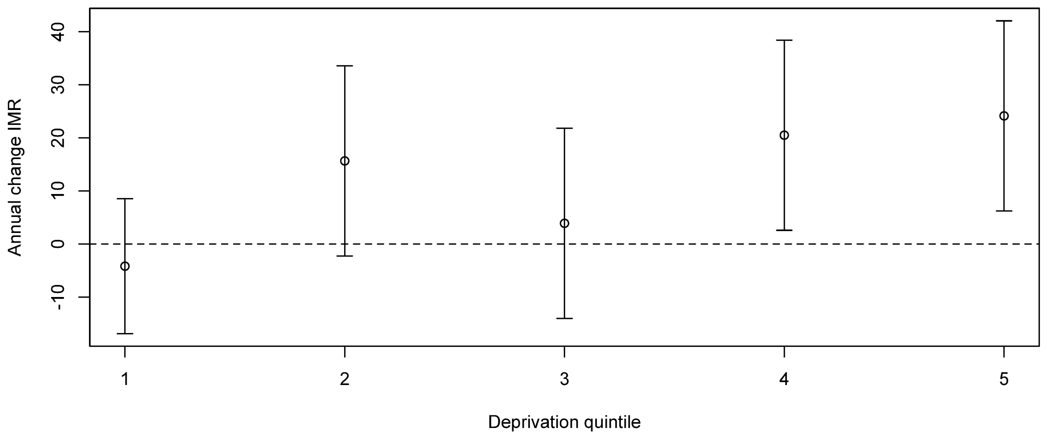

Taylor-Robinson D, *et al. BMJ Open* 2019; 9:e029424. doi: 10.1136/bmjopen-2019-029424

Web appendix 10: Neonatal mortality and post-neonatal mortality trend by deprivation quintile of local authority district, 2000-2017, with 95% binomial confidence intervals.

A neonatal death is defined as a death during the first 28 days of life (0-27 days); post-neonatal between 28 and 364 days after birth.

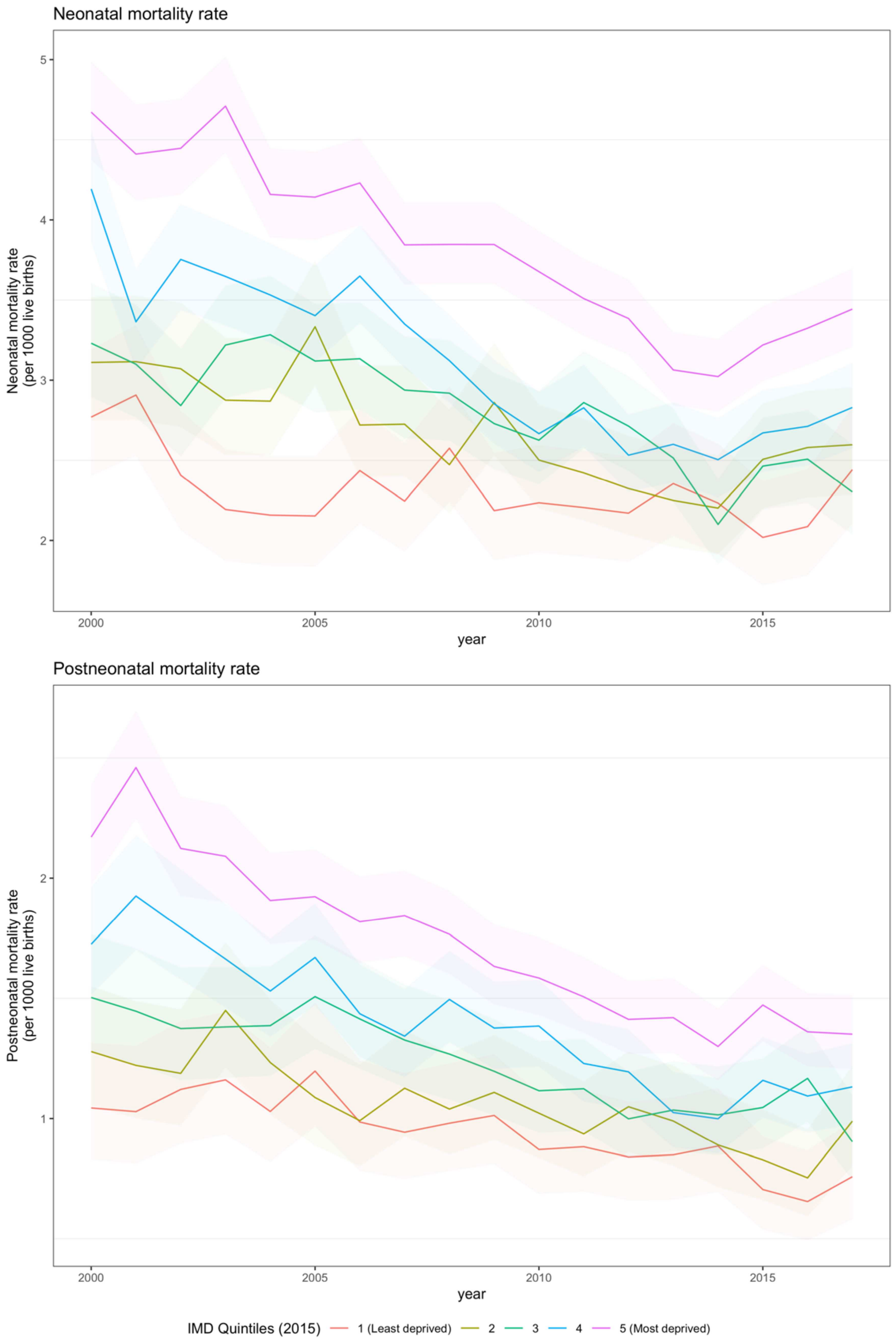

Taylor-Robinson D, *et al. BMJ Open* 2019; 9:e029424. doi: 10.1136/bmjopen-2019-029424

Web appendix 11: Estimates from the main analysis repeated using neonatal mortality and post-neonatal mortality as the outcome, plotted with their confidence intervals.

Estimates for annual change (deaths per 100,000) in neonatal mortality and post-neonatal mortality 2014-2017 relative to previous trend (2000-2013) and their confidence intervals by small area deprivation quintile (most deprived=quintile 5).

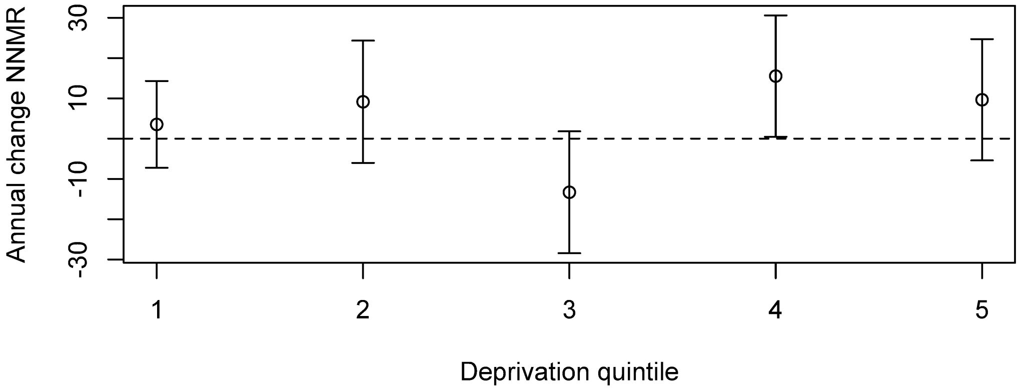

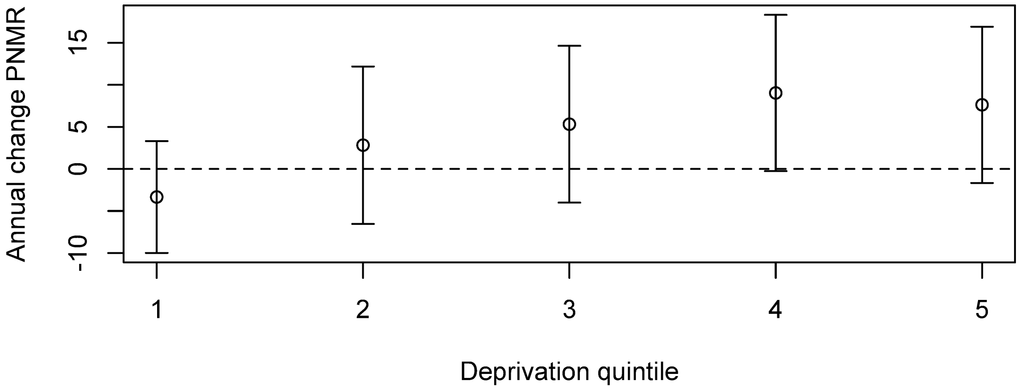

Taylor-Robinson D, *et al. BMJ Open* 2019; 9:e029424. doi: 10.1136/bmjopen-2019-029424
