# Supplementary material for: Assessing the impact of rising child poverty on the unprecedented rise in infant mortality in England, 2000–2017: time trend analysis
Source: BMJ Open. 2019 Oct 2;9(10):e029424. doi: 10.1136/bmjopen-2019-029424 (PMC6954495; doi:10.1136/bmjopen-2019-029424)
Supplement: Author's manuscript [file bmjopen-2019-029424.draft_revisions.pdf]

BMJ Open is committed to open peer review. As part of this commitment we make the peer review history of every article we publish publicly available.

When an article is published we post the peer reviewers' comments and the authors' responses online. We also post the versions of the paper that were used during peer review. These are the versions that the peer review comments apply to.

The versions of the paper that follow are the versions that were submitted during the peer review process. They are not the versions of record or the final published versions. They should not be cited or distributed as the published version of this manuscript.

BMJ Open is an open access journal and the full, final, typeset and author-corrected version of record of the manuscript is available on our site with no access controls, subscription charges or pay-per-view fees (http://bmjopen.bmj.com).

If you have any questions on BMJ Open's open peer review process please email info.bmjopen@bmj.com

BMJ Open

Child poverty and the unprecedented rise in infant mortality in England 2000-17: time trend analysis

Journal:	*BMJ Open*
Manuscript ID	bmjopen-2019-029424
Article Type:	Research
Date Submitted by the Author:	25-Jan-2019

Complete List of Authors:	Taylor-Robinson, David; University of Liverpool, Department of Public Health and Policy<br>Lai, Eric; University of Liverpool, Public Health and Policy<br>Wickham, S; University of Liverpool<br>Rose, Tanith; University of Liverpool, Public Health and Policy<br>Norman, Paul; University of Leeds, School of Geography<br>Bambra, Clare; Newcastle University, Institute of Health & Society<br>Whitehead, Margaret; University of Liverpool, Public Health<br>Barr, Ben ; University of Liverpool, Department Public Health and Policy

Keywords:	EPIDEMIOLOGY, PUBLIC HEALTH, Community child health < PAEDIATRICS, Health policy < HEALTH SERVICES ADMINISTRATION & MANAGEMENT

SCHOLARONE™ Manuscripts

Child poverty and the unprecedented rise in infant mortality in England 2000-17: time trend analysis

David Taylor-Robinson[1], Eric TC Lai[1], Sophie Wickham[1], Tanith Rose[1], Paul Norman[2], Clare Bambra[3], Margaret Whitehead[1], Ben Barr[1]

1. Department of Public Health and Policy, The Farr Institute@HeRC, Waterhouse Building 2nd Floor Block F, University of Liverpool, Liverpool, United Kingdom, L69 3GB.
2. School of Geography & Leeds Institute of Data Analytics, University of Leeds, Woodhouse Lane, Leeds, LS2 9JT
3. Institute of Health & Society, Baddiley-Clark, Richardson Road, Newcastle upon Tyne, NE2 4AX

Corresponding author: Professor David Taylor-Robinson; email: dctr@liv.ac.uk

For peer review only

For peer review only - http://bmjopen.bmj.com/site/about/guidelines.xhtml

Abstract

Objective

To determine whether there were inequalities in the sustained rise in infant mortality in England in recent years and the contribution of rising child poverty to these trends.

Design

Analysis of trends in infant mortality in local authorities grouped by quintiles of income deprivation. Fixed effects regression models were used to quantify the association between regional changes in child poverty and regional changes in infant mortality.

Setting

324 English local authorities in 9 English Government Office Regions.

Participants

Live born children under 1 year of age.

Main outcome measure

Infant mortality rate (IMR), defined as the number of deaths in children under 1 year of age per 100,000 live births in the same year.

Results

The sustained and unprecedented rise in infant mortality in England from 2014-2017 was not experienced evenly across the population. In the most deprived local authorities, the previously declining trend in infant mortality reversed and mortality rose, leading to an additional 24 infant deaths per 100,000 live births per year (95% CI 6-42), relative to the previous trend. There was no significant change from the pre-existing trend in the most affluent local authorities, increasing inequalities. Overall from 2014-2017, there were a total of 572 excess infant deaths (95% CI 200 to 944) compared to expected historical trends. Each 1% increase in child poverty was significantly associated with an extra 5.8 infant deaths per 100,000 live births (95%CI 2.4 to 9.2). About a third of the increases in infant mortality between 2014 and 2017 can be attributed to rising child poverty (172 deaths 95%CI 74 to 266).

For peer review only - http://bmjopen.bmj.com/site/about/guidelines.xhtml

For peer review only

Conclusion

The unprecedented rise in infant mortality disproportionately affected the poorest areas of the country, leaving the more affluent areas unaffected. About a third of the increase in infant mortality from 2014-17 may be attributed to rising child poverty.

For peer review only

For peer review only - http://bmjopen.bmj.com/site/about/guidelines.xhtml

Strengths and limitations of this study

This is the first study to explore rising infant mortality rates in England; whether this increase affected all sections of the population evenly; and how much can be attributed to rising levels of child poverty.

A strength is the use of the most up-to date infant mortality and child poverty data over a 17-year time period, using fixed-effects methods to adjust for confounding by unobserved factors.

A limitation is that current measures of child poverty in England underestimate the extent of poverty and are a simplification of more complex underlying trajectories.

For peer review only

Introduction

Infant mortality rate (IMR) has risen for the last four years in England, yet the role of increasing levels of child poverty in explaining these trends is unclear. Along with others[1], we raised concern about recent rises in infant mortality in England in two letters to the BMJ.[2,3] We noted that this rise had occurred particularly amongst more disadvantaged children from routine and manual socioeconomic groups. Rising infant mortality is unusual in high income countries, and international data show that infant mortality has continued to decline in most rich countries in recent years.[4] Infant mortality is a sensitive indicator of the changing overall health of societies and, as such, acts as an early warning system for future adverse trends. There is therefore an urgent need to understand this extremely concerning trend in England.

Adverse trends in mortality have occurred across all age groups in England in recent years.[5] Several commentators have suggested that these could be due to austerity policies introduced in recent years including cuts to NHS, local authority and public health services and changes to welfare benefits. [6,7] Since 2010, there have been sustained reductions in the welfare benefits available to families with children including: the abolition of child benefit and child tax credit for the third child or more; reductions in the value of tax credits, below inflation up-rating of most working-age benefits; housing benefit reforms including the under occupancy charge (most commonly referred to as 'bedroom tax'), introduction of universal credit; and household caps on total benefit receipt (regardless of how many children are in the household).[8] These welfare changes have disproportionately affected the most deprived local authorities and regions [8] and have led to a rise in child poverty.[9] The impacts of these changes on trends in child health have not been considered. For example, a recent review of mortality increases by Public Health England did not consider the potential causes of increases in infant mortality.[5]

There is strong evidence that increased child poverty leads to deteriorating child health and increased infant deaths[10–14]. Whilst relative child poverty declined between 2007 and 2013, we are now seeing increases: child poverty (defined as living in a household with income below 60% of the median household after housing costs) rose by 2 percentage points between 2014 and 2017 and it is projected to increase further through to 2022.[9] By 2017

For peer review only - http://bmjopen.bmj.com/site/about/guidelines.xhtml

there were 4.1 million children in England living in relative poverty amounting to 30% of all English children. This compares to less than 10% of children in European countries such as Austria, Denmark, Finland, Iceland, Norway, Slovenia, Sweden, and Switzerland.[15] The recent rises in infant mortality in England have occurred concurrently with these increases in child poverty (figure 1).

Figure 1: Trends in infant mortality and child poverty in England, 2007-17

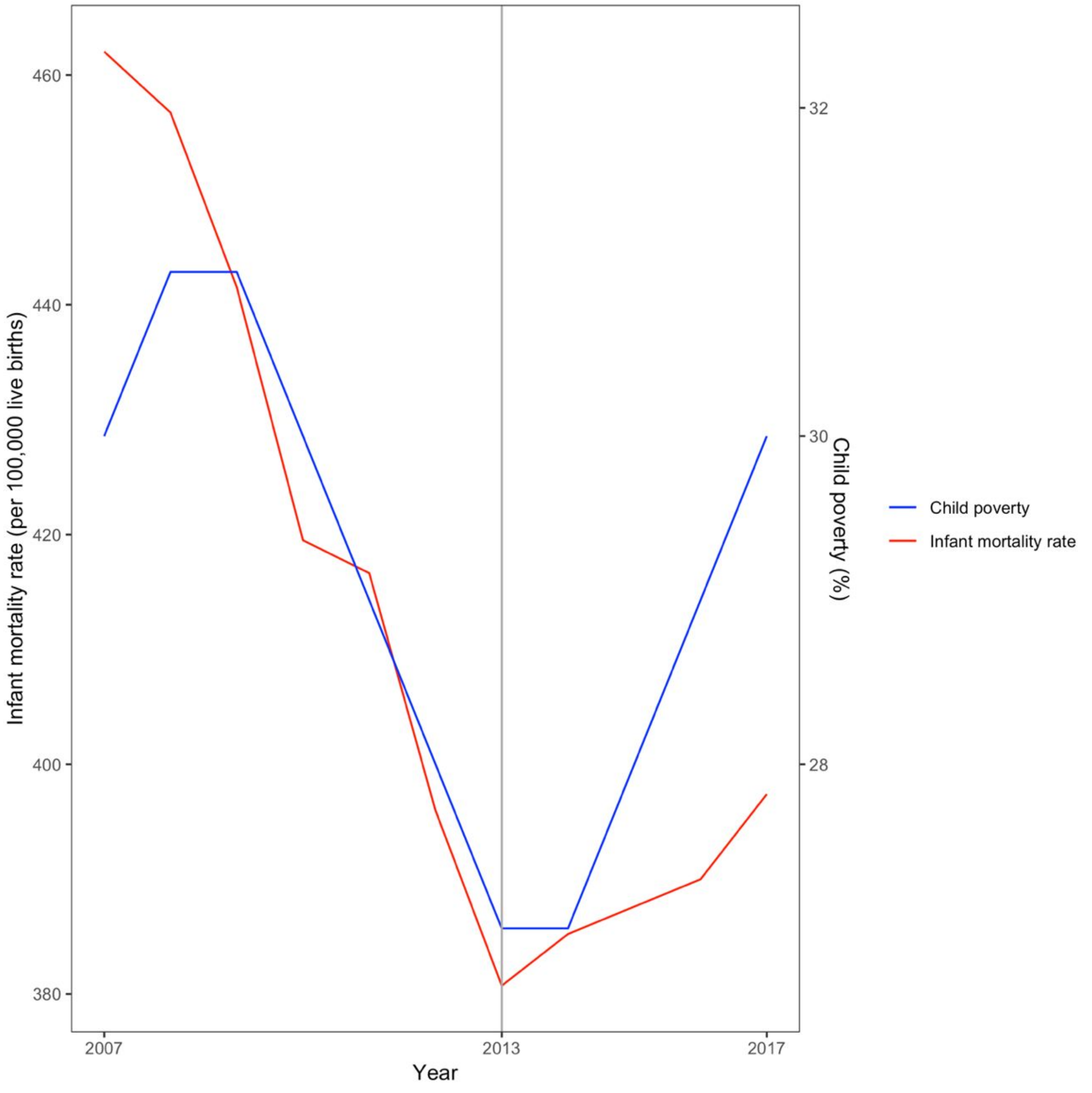

In this paper, we investigate whether there were inequalities in the sustained rise in infant mortality in England in recent years and the contribution of rising child poverty to these trends. Firstly, we investigate whether infant mortality increased more in those parts of the

For peer review only - http://bmjopen.bmj.com/site/about/guidelines.xhtml

country with the highest numbers of people receiving low income related welfare benefits, as these are the areas that are most likely to have been adversely affected by recent changes in welfare policy. We estimate whether there was a significant change in the trend in infant mortality in those areas and the timing of this change. Secondly, we exploit regional differences in trends in infant mortality and child poverty to estimate the extent to which those regions with greater increases in child poverty experienced greater increases in infant mortality. We use these estimates to calculate the proportion of recent infant mortality increases that are potentially explained by increases in child poverty.

Methods

Annual Vital Statistics data for the number of registered infant deaths (< 1 year of age) and live births for 324 lower tier local authority level (324 local areas) between 2000 to 2017 were obtained from the Office of National Statistics. Local authorities were grouped in 5 categories (quintiles) based on the income deprivation score of the 2015 Indices of Multiple Deprivation. This score is a non-overlapping count of the numbers of people in each local authority on a low income and in receipt of means tested benefits as a proportion of the population.[16]

Regional relative child poverty was measured as the proportion of children living in households in each region with less than 60 per cent of contemporary national median household income, using the Households Below Average Income data provided by the Department for Work and Pensions.[17] This is the most widely used measure of poverty within the European Union and recognises that the experience of poverty is relative to standards of living that are considered normal within a society and that these average standards change over time. [14] As this measure not available at the local authority level we used data for the nine government office regions of England. [18]

Statistical analysis

The statistical analysis proceeded in three steps. Firstly, we assessed descriptive trends in infant mortality between 2000 and 2017 for the five groups of local authorities categorised by their level of income deprivation (see above). Secondly, we tested whether there was a statistically significant change in the infant mortality trend during this time, the timing of that

For peer review only - http://bmjopen.bmj.com/site/about/guidelines.xhtml

change in trend and whether any change in trend differed by the level of income deprivation. To do this we used longitudinal local authority area level data to estimate a segmented mixed effects regression model for infant mortality including two linear spline terms for time interacted with income deprivation quintile, with a breakpoint indicating the change in trend. We used an iterative search procedure to identify which breakpoint provided the best fit for the data, [19,20] in other words identifying at which time point a change in trend occurred. This model included random effects (intercepts) at the local authority level to take account of the longitudinal nature of the data (see appendix for details). Secondly, we used this model to estimate whether there had been a significant change in trend in recent years, whether this had led to widening inequalities between local authority areas based on their level of income deprivation and to estimate the level of excess mortality that was attributable to any change in trend. Excess mortality was estimated as the marginal difference between the observed trend and that which would be predicted from the model if pre-existing trends had continued, using the *margins[21]* commands in Stata version 14

In the third step of the analysis, we assessed the association of changes in regional child poverty and regional infant mortality. As there is potential confounding from unobserved factors that vary between regions, or national trends in unobserved factors that affect all region, we used a fixed effects approach to remove between region differences and national trends.[22,23] This conservative approach involves including dummy variables for each region and year to assess the association between deviations from the average rate of change in poverty and deviations from average rate of change in infant mortality in each region (see appendix for details). This method means that the estimated association between child poverty and infant mortality cannot be confounded by any time invariant differences between regions or any national trend that effects all regions, providing an estimate that is likely to reflect a causal association. We aligned annual regional child poverty rates for financial years to infant mortality registered in the calendar year in which the financial year ended. In other words, 2016-17 child poverty data was aligned with 2017 infant mortality data, meaning that child poverty measures were lagged by 9 months compared to infant mortality measures. We used robust clustered standard errors to ensure that they are robust to serial correlation in the data. This model was used to estimate the number of child deaths attributable to child poverty increases in recent years, by estimating the marginal difference between the observed trend and that which would be predicted from the model if child poverty had not increased *[21]*. All models were estimated using Stata version 14 and R version 3.5.1.

For peer review only - http://bmjopen.bmj.com/site/about/guidelines.xhtml

Results

Infant mortality had been falling for all groups of local authority areas since 2000, with greater decreases in the most income deprived areas, reducing inequalities. From 2013, this trend changed, and infant mortality increased particularly in the most income deprived local authority areas (figure 2).

Figure 2: Infant mortality trend by deprivation quintile of local authority district, 2000-2017, with 95% binomial confidence intervals.

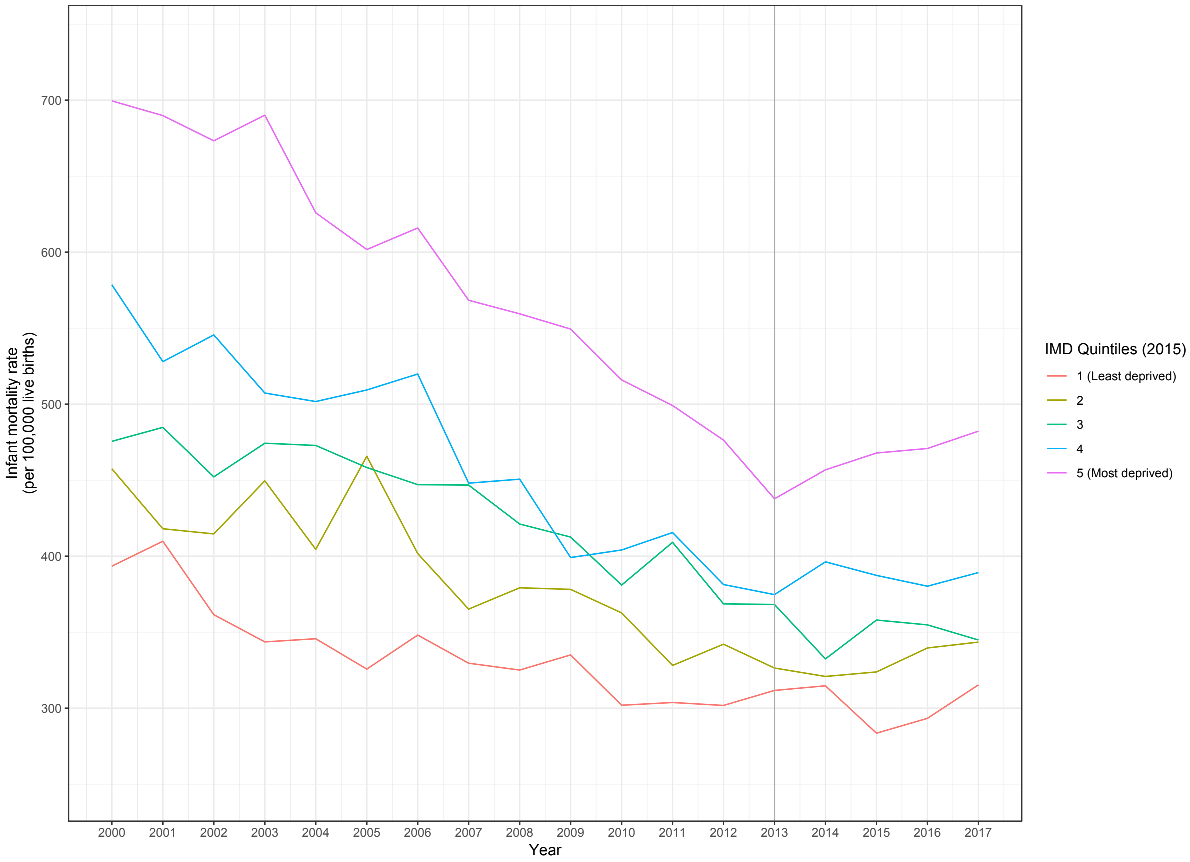

The breakpoint analysis also showed a significant change in trend from 2013 (appendix). Table 1 shows the results of the regression model for trends in infant mortality since that time. From 2013-17 there was a dramatic reversal of the pre-existing trend, with an additional annual increase of 24 deaths per 100,000 live births (95%CI 6 to 42) in the most deprived quintile 5 compared to the 2000-2013 trend. A significant change in trend was also observed for quintile 4, whilst there was no significant change from the previously declining trend in the most affluent quintile. The gap in infant mortality between the most income

For peer review only - http://bmjopen.bmj.com/site/about/guidelines.xhtml

deprived and most affluent quintile increased by 52 deaths per 100,000 births (95% CI 36 to 68). Based upon these trends we estimated that there were an additional 570 excess deaths (95% CI 200 to 944) in the period 2014-17 than would have been expected if the historical trends had continued.

Table 1: Segmented regression model results for trends in infant mortality at lower tier local authority level 2014-2017 (per 100,000 live births per year)

Level of income deprivation	Annual change (deaths per 100,000) in infant mortality 2014-2017 relative to previous trend (2000-2013) (95% CI)		P value for change in trend from previous period
Quintile 1 (most affluent)	-4.19	[-16.91,8.53]	0.518
Quintile 2	15.65	[-2.27,33.57]	0.087
Quintile 3	3.89	[-14.03,21.81]	0.67
Quintile 4	20.5	[2.58,38.42]	0.025
Quintile 5 (most deprived)	24.14	[6.22,42.05]	0.008

Estimates based on random effects regression model using LA panel dataset of infant mortality from 2000 to 2017, N= 5,832 local authority years.

In the fixed effects analysis each percentage point change in child poverty was associated with a change in infant mortality of 5.8 deaths per 100,000 live births (95% confidence interval 2.4 to 8.9). From this model we estimate that 172 deaths 95% CI (74 to 266) between 2014 and 2017 were attributable to increases in child poverty, almost a third of the overall rise in infant mortality over that period.

Discussion

Since 2013, infant mortality has increased in England and there have been an additional 570 infant deaths over 4 years (2014 to 2017) compared to what would have been expected based on recent historical trends. These excess deaths have largely occurred in the most disadvantaged areas, increasing inequalities. Our study estimated that the recent increase in levels of child poverty was associated with 172 (about a third) of the extra infant deaths in England in the period 2014-17.

For peer review only - http://bmjopen.bmj.com/site/about/guidelines.xhtml

For peer review only

Limitations of the study

Before evaluating the implications of our findings, we note several important limitations. Firstly, although we undertook an observational analysis at the population level, lack of data on levels of child poverty at a small area level meant that we undertook our analysis of the effects of child poverty using child poverty estimates for 9 regions within England. This meant we could take advantage of regional differences in trends over time, and use a fixed effects approach to adjust for time invariant confounding variables. We were not able, however, to estimate the effect of increases in child poverty on trends in infant mortality for groups of local authorities within these regions. As our main analysis was based on aggregate data, however, we cannot identify whether the additional infant deaths were in the same families who have experienced a rise in child poverty. It is also possible that the association between the child poverty and infant mortality in our analysis was due to trends in unobserved time-varying confounding factors that varied between regions. However, the strong evidence that increased child poverty leads to deteriorating child health and increased infant deaths makes it highly plausible that the association is causal. [10–14]

Secondly, we measured child poverty using the relative poverty measure used by the Department for Work and Pensions in England. We used this measure because it is used internationally, as well as in the UK, and is of current policy relevance and debate.[24] This measure, however, is a simplification of underlying income trajectories whereby families with children move in and out of poverty over time. Furthermore, the measure we have used is likely to underestimate the extent of the experience of poverty in England. A recent report published by the Social Metrics Commission outlining a new measure of poverty which extends beyond income-based poverty, suggests that there are 4.5 million children living (32.6%) in poverty in the UK.[25] In addition, the Joseph Rowntree Foundation's (JRF) has shown that the proportion of children living below the Minimum Income Standard has increased from 39% to 45% (an increase of about 1 million) between 2008-09 and 2014-15.[26]

Thirdly, we were not able to investigate the factors mediating the association between rising child poverty and rising infant mortality, such as birth characteristics (for example preterm birth, maternal age and maternal smoking) and post-natal factors (for example breastfeeding and child care in the first year of life). [27,28]

For peer review only - http://bmjopen.bmj.com/site/about/guidelines.xhtml

Comparison with previous studies

To our knowledge this is the first study to explore both the inequalities in the unprecedented rise in infant mortality in England on the basis of area deprivation and the reasons behind this rise. Whilst the UK government recently commissioned a report into adverse mortality trends, infant mortality was not considered in any detail. [5] Our analysis corroborates our previous analysis of trends in infant mortality on the basis of parental occupational social class, which suggested that IMR was rising particularly in the most disadvantaged social groups.[3,29] A recent report by the Royal College of Paediatrics and Child Health (RCPCH) also noted the reversal of over 100 year of declining infant mortality in England and Wales, and that rises in infant mortality were most striking in the deprived portion of the population. [27] The RCPCH further assesses various scenarios of infant mortality, for instance showing that even if infant mortality begins to decline again at its previous rate, rates will be 80% higher than the EU15+ average in 2030. Our study uses the most up-to date infant mortality data, and suggests that rather than just stalling, infant mortality is rising sharply in the most disadvantaged areas.

In addition to increasing inequalities in infant mortality, social inequalities in numerous aspects of health in England appear to be rising.[7,19,30,31] Barr and colleagues demonstrated a reversal in trends in inequalities in life expectancy.[19] Two analyses from the Global Burden of Diseases collaboration have shown both a stagnation in the improvement in life expectancy in England; and increasing inequalities since 2010.[30,31] Furthermore Bennett et al identify deaths in children younger than 5 years as a major contributor to inequality in life expectancy.[30] A further analysis has demonstrated an increase in the North-South divide in health in England.[7]

Our study shows that rising income poverty may be contributing to rising infant mortality. Numerous studies conducted in Western Europe and the USA have shown an association between social disadvantage and infant mortality. [32–35] Furthermore, a recent systematic review by Cooper and Stewart demonstrated clear causal links between income poverty and a range of child health outcomes, including only studies that applied quasi-experimental designs.[13,36] Komro and colleagues, for example, used a difference-in-differences research design to assess the impact of state-level minimum wage in the USA on infant

For peer review only - http://bmjopen.bmj.com/site/about/guidelines.xhtml

mortality. The authors show that each dollar increase in the minimum wage above the federal level was associated with a 1% to 2% decrease in low birth weight births and a 4% decrease in postneonatal mortality. [37] Additionally, the results of a longitudinal analysis of social welfare expenditure data from 19 Organisation for Economic Co-operation and Development (OECD) countries over the time period from 1980 to 2010, showed that cash benefits for families have positive effects on reducing infant mortality, with a $10 increase in family cash allowances per child predicting a reduction in infant mortality by 4% (p = .007).[12] Previous research has shown that expansions to social security nets and public services have decreased inequalities in life expectancy [19], infant mortality rates [38], and mortality amenable to healthcare.[39] Robinson et al, for example, found that the English health inequalities strategy (2000-2010) was associated with decreases in inequalities in infant mortality in England. [38] Similarly, Krieger et al found that the 1960s 'War on Poverty' led to decreases in inequalities in IMR in the USA.[40]

Policy implications

Our analysis has important implications in the context of the projected ongoing increase in levels of child poverty in the UK. For the first time in nearly 40 years there has been a sustained increase in infant mortality in the poorest areas. English regions with the largest rises in child poverty have had the largest increases in infant mortality. Our analysis suggests that the weakened social protection safety net - leading to rising levels of child poverty – may be contributing to this rise in mortality in the most disadvantaged infants. Our analysis suggests that the rise in child poverty explains approximately a third of this rise. This could be an underestimate of the impact if the measures of child poverty used are an underestimate of the true increase in disadvantage facing children (see above). It may also be the case that other policy changes occurring at this time such as the real terms cuts to NHS, local authority children's services, social care and public health budgets –are also be contributing to the rise in mortality as has been suggested elsewhere. [6,7]

Infant mortality is an uncommon event and represents the most severe tip of the iceberg in terms of the impact of social conditions on health. It can, however, act as an early warning system or litmus test of the overall health of societies. Rising poverty is likely to be having a myriad of adverse impacts on other aspects of child health that will have repercussions across

For peer review only - http://bmjopen.bmj.com/site/about/guidelines.xhtml

the lifecourse.[36] In the context of increasing health inequalities in England, policies that reduce poverty and social inequalities, investing in child health and social care are likely to reduce the occurrence of infant mortality and that of many other adverse child health outcomes.[30]

This rise in mortality in the most disadvantaged children is unprecedented and requires urgent action by national and local governments and the health and social care system. It is likely that the rise in child poverty is an important factor contributing to this trend. As the United Nations (UN) has recently highlighted, rising poverty in the UK is a political choice[41] and it is time for the government to reverse this trend establishing a welfare system that protects children from poverty.

For peer review only

For peer review only - http://bmjopen.bmj.com/site/about/guidelines.xhtml

Funding: DTR and EL are funded by the MRC on a Clinician Scientist Fellowship (MR/P008577/1). SW is supported by a Wellcome Trust Society and Ethics fellowship (grant number 200335/Z/15/Z). BB and TR are supported by the National Institute for Health Research (NIHR) Collaboration for Leadership in Health Research and Care (CLAHRC NWC).

The MRC, Wellcome Trust and NIHR had no role in the study design, data collection and analysis, decision to publish, or preparation of the manuscript. This report is independent research arising from research supported by the MRC, Wellcome Trust and NIHR. The views expressed in this publication are those of the authors and not necessarily those of the NHS, the NIHR, or the Department of Health and Social Care.

For peer review only

Competing interest statement

All authors have completed the ICMJE uniform disclosure format www.icmje.org/coi_disclosure.pdf and declare: DTR and EL are funded by the MRC on a Clinician Scientist Fellowship (MR/P008577/1). SW is supported by a Wellcome Trust Society and Ethics fellowship (grant number 200335/Z/15/Z). BB and TR are supported by the National Institute for Health Research (NIHR) Collaboration for Leadership in Health Research and Care (CLAHRC NWC); no financial relationships with any organisations that might have an interest in the submitted work in the previous three years; no other relationships or activities that could appear to have influenced the submitted work.

Contributorship statement

DTR is lead author and guarantor. DTR and BB planned the study and led the drafting and revising of the manuscript. DTR, BB and EL conducted the analysis. DTR, BB, EL, SW, TR, PN, CB and MMW contributed to interpretation of the data, drafting of the manuscript, and revisions. All authors agreed the submitted version of the manuscript.

Transparency declaration

DTR is the guarantor and affirms that the manuscript is an honest, accurate, and transparent account of the study being reported; that no important aspects of the study have been omitted;

For peer review only - http://bmjopen.bmj.com/site/about/guidelines.xhtml

and that any discrepancies from the study as planned (and, if relevant, registered) have been explained.

Ethics committee approval

None required

Data sharing

Statistical code available on request from lead author

Licence statement

The Corresponding Author has the right to grant on behalf of all authors and does grant on behalf of all authors, a worldwide licence (http://www.bmj.com/sites/default/files/BMJ%20Author%20Licence%20March%202013.doc) to the Publishers and its licensees in perpetuity, in all forms, formats and media (whether known now or created in the future), to i) publish, reproduce, distribute, display and store the Contribution, ii) translate the Contribution into other languages, create adaptations, reprints, include within collections and create summaries, extracts and/or, abstracts of the Contribution and convert or allow conversion into any format including without limitation audio, iii) create any other derivative work(s) based in whole or part on the on the Contribution, iv) to exploit all subsidiary rights to exploit all subsidiary rights that currently exist or as may exist in the future in the Contribution, v) the inclusion of electronic links from the Contribution to third party material where-ever it may be located; and, vi) licence any third party to do any or all of the above. All research articles will be made available on an open access basis (with authors being asked to pay an open access fee—see http://www.bmj.com/about-bmj/resources-authors/forms-policies-and-checklists/copyright-open-access-and-permission-reuse). The terms of such open access shall be governed by a Creative Commons licence—details as to which Creative Commons licence will apply to the research article are set out in our worldwide licence referred to above.

For peer review only - http://bmjopen.bmj.com/site/about/guidelines.xhtml

References

1 Hiam L, Dorling D. Government's misplaced prevention agenda. *BMJ* 2018;**363**:k5134. doi:10.1136/bmj.k5134

2 Taylor-Robinson D, Barr B. Death rate now rising in UK's poorest infants. *BMJ* 2017;**357**:j2258. doi:10.1136/bmj.j2258

3 Death rate continues to rise for poorest infants in England and Wales. *The BMJ* Published Online First: 13 September 2018.https://www.bmj.com/content/360/bmj.k1090/rr-2 (accessed 17 Sep 2018).

4 Health status - Infant mortality rates - OECD Data. theOECD. http://data.oecd.org/healthstat/infant-mortality-rates.htm (accessed 23 Nov 2018).

5 Public Health England. A review of recent trends in mortality in England. London: : PHE 2018.

6 Bennett JE, Li G, Foreman K, *et al.* The future of life expectancy and life expectancy inequalities in England and Wales: Bayesian spatiotemporal forecasting. *The Lancet* 2015;**386**:163–70. doi:10.1016/S0140-6736(15)60296-3

7 Kontopantelis E, Buchan I, Webb RT, *et al.* Disparities in mortality among 25–44-year-olds in England: a longitudinal, population-based study. *The Lancet Public Health* 2018;**0**. doi:10.1016/S2468-2667(18)30177-4

8 Beatty C, Fothergill S. The uneven impact of welfare reform : the financial losses to places and people. 2016. doi:10.7190/cresr.2017.5563239352

9 Hood A, Waters T, Institute for Fiscal Studies (Great Britain), *et al. Living standards, poverty and inequality in the UK: 2016-17 to 2021-22*. 2017.

10 Wang H, Liddell CA, Coates MM, *et al.* Global, regional, and national levels of neonatal, infant, and under-5 mortality during 1990–2013: a systematic analysis for the Global Burden of Disease Study 2013. *The Lancet* 2014;**384**:957–79. doi:10.1016/S0140-6736(14)60497-9

11 Zylbersztejn A, Gilbert R, Hjern A, *et al.* Child mortality in England compared with Sweden: a birth cohort study. *The Lancet* 2018;**0**. doi:10.1016/S0140-6736(18)30670-6

12 Shim J. Social Welfare Expenditures and Infant Mortality. *Soc Work Public Health* 2015;**30**:567–77. doi:10.1080/19371918.2015.1073648

13 Cooper K, Stewart K. *Does Money Affect Children's Outcomes?: A Systematic Review*. Joseph Rowntree Foundation 2013.

14 Wickham S, Anwar E, Barr B, *et al.* Poverty and child health in the UK: using evidence for action. *Archives of disease in childhood* Published Online First: 2016. doi:10.1136/archdischild-2014-306746

15 OECD. Child_Poverty. https://www.oecd.org/els/CO_2_2_Child_Poverty.pdf (accessed 17 Dec 2018).

For peer review only - http://bmjopen.bmj.com/site/about/guidelines.xhtml

16 English indices of deprivation 2015. GOV.UK. https://www.gov.uk/government/statistics/english-indices-of-deprivation-2015 (accessed 10 Dec 2018).

17 Households below average income (HBAI) statistics. GOV.UK. https://www.gov.uk/government/collections/households-below-average-income-hbai--2 (accessed 23 Nov 2018).

18 ONS. Administrative geography. https://www.ons.gov.uk/methodology/geography/ukgeographies/administrativegeography/england (accessed 14 Dec 2018).

19 Barr B, Higgerson J, Whitehead M. Investigating the impact of the English health inequalities strategy: time trend analysis. *BMJ* 2017;**358**:j3310. doi:10.1136/bmj.j3310

20 Crawley M. *An Introduction to Statistics using R*. Chichechester: : John Wiley and Sons 2005.

21 Marginal analysis | Stata. https://www.stata.com/features/overview/marginal-analysis/ (accessed 18 Dec 2018).

22 Barr B, Taylor-Robinson D, Scott-Samuel A, *et al.* Suicides associated with the 2008-10 economic recession in England: time trend analysis. *BMJ* 2012;**345**:e5142. doi:10.1136/bmj.e5142

23 Barr B, Taylor-Robinson D, Stuckler D, *et al.* 'First, do no harm': are disability assessments associated with adverse trends in mental health? A longitudinal ecological study. *J Epidemiol Community Health* 2015;:jech-2015-206209. doi:10.1136/jech-2015-206209

24 FPH. APPG Inquiry: Child Poverty and Health. https://www.fph.org.uk/media/1374/appg-health-in-all-policies-inquiry-into-child-poverty-and-health2.pdf (accessed 14 Dec 2018).

25 Stroud P. The final report of the Social Metrics Commission: A new measure of poverty for the UK. https://lif.blob.core.windows.net/lif/docs/default-source/default-library/legj6470-measuring-poverty-full_report-181004-web.pdf?sfvrsn=0

26 Households below a Minimum Income Standard: 2008/09 to 2014/15. JRF. 2017.https://www.jrf.org.uk/report/households-below-minimum-income-standard-200809-201415 (accessed 10 Dec 2018).

27 Child health in England in 2030: comparisons with other wealthy countries | RCPCH. https://www.rcpch.ac.uk/resources/child-health-england-2030-comparisons-other-wealthy-countries (accessed 10 Dec 2018).

28 Sidebotham P, Fraser J, Covington T, *et al.* Understanding why children die in high-income countries. *Lancet* 2014;**384**:915–27. doi:10.1016/S0140-6736(14)60581-X

29 Taylor-Robinson D, Barr B. Death rate now rising in UK's poorest infants. *BMJ* 2017;**357**:j2258. doi:10.1136/bmj.j2258

For peer review only

For peer review only - http://bmjopen.bmj.com/site/about/guidelines.xhtml

30 Bennett JE, Pearson-Stuttard J, Kontis V, *et al.* Contributions of diseases and injuries to widening life expectancy inequalities in England from 2001 to 2016: a population-based analysis of vital registration data. *The Lancet Public Health* 2018;**3**:e586–97. doi:10.1016/S2468-2667(18)30214-7

31 Steel N, Ford JA, Newton JN, *et al.* Changes in health in the countries of the UK and 150 English Local Authority areas 1990–2016: a systematic analysis for the Global Burden of Disease Study 2016. *The Lancet* 2018;**392**:1647–61. doi:10.1016/S0140-6736(18)32207-4

32 Kim D, Saada A. The social determinants of infant mortality and birth outcomes in Western developed nations: a cross-country systematic review. *Int J Environ Res Public Health* 2013;**10**:2296–335. doi:10.3390/ijerph10062296

33 Weightman AL, Morgan HE, Shepherd MA, *et al.* Social inequality and infant health in the UK: systematic review and meta-analyses. *BMJ Open* 2012;**2**:e000964. doi:10.1136/bmjopen-2012-000964

34 Gisselmann MD. The influence of maternal childhood and adulthood social class on the health of the infant. *Soc Sci Med* 2006;**63**:1023–33. doi:10.1016/j.socscimed.2006.03.015

35 Kihal-Talantikite W, Padilla CM, Lalloué B, *et al.* Green space, social inequalities and neonatal mortality in France. *BMC Pregnancy Childbirth* 2013;**13**:191. doi:10.1186/1471-2393-13-191

36 Cooper K, Stewart K. Money Matters: Does Money Affect Children's Outcomes? A Systematic Review Update. http://sticerd.lse.ac.uk/case/_new/research/money_matters/default.asp

37 Komro KA, Livingston MD, Markowitz S, *et al.* The Effect of an Increased Minimum Wage on Infant Mortality and Birth Weight. *Am J Public Health* 2016;**106**:1514–6. doi:10.2105/AJPH.2016.303268

38 Robinson TJ, Barr B, Brown HW, *et al.* OP18 Evaluating the impact of the english health inequalities strategy on socioeconomic inequalities in the regional infant mortality rate. *J Epidemiol Community Health* 2018;**72**:A9–A9. doi:10.1136/jech-2018-SSMabstracts.18

39 Barr B, Bambra C, Whitehead M. The impact of NHS resource allocation policy on health inequalities in England 2001-11: longitudinal ecological study. *BMJ* 2014;**348**:g3231. doi:10.1136/bmj.g3231

40 Krieger N, Rehkopf DH, Chen JT, *et al.* The fall and rise of US inequities in premature mortality: 1960-2002. *PLoS Med* 2008;**5**:e46. doi:10.1371/journal.pmed.0050046

41 Poverty is a political choice. *The Lancet Public Health* 2018;**3**:e555. doi:10.1016/S2468-2667(18)30243-3

For peer review only

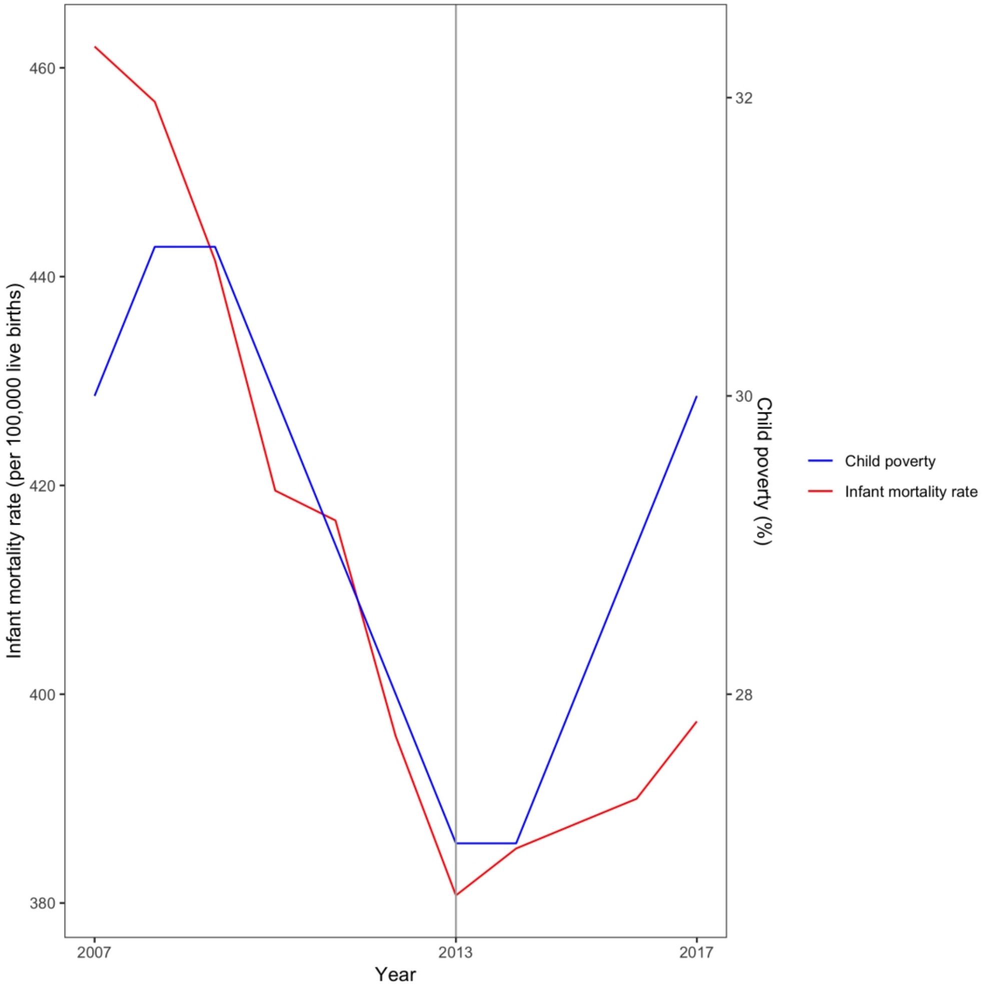

833x833mm (72 x 72 DPI)

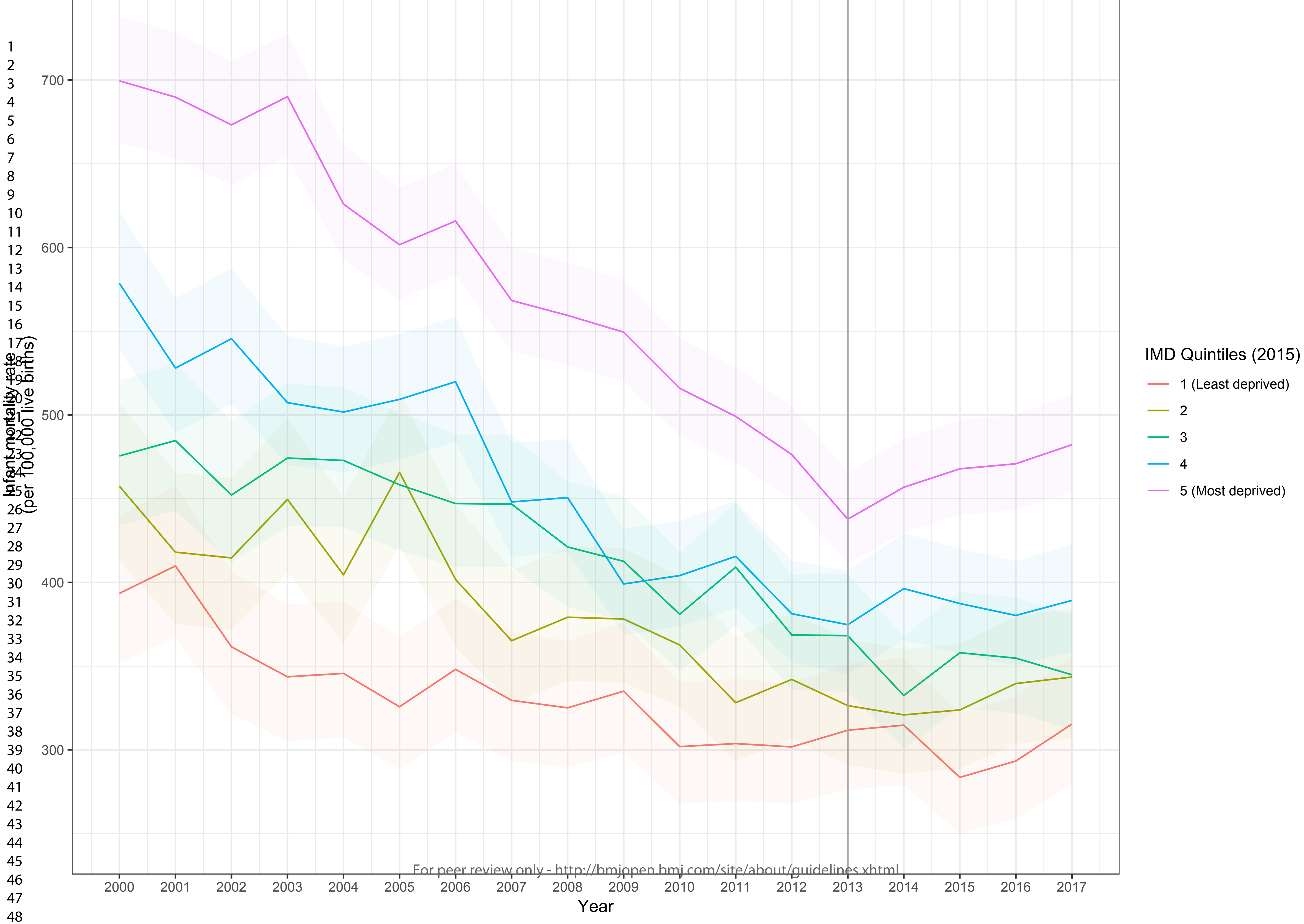
Infant mortality rate
(per 100,000 live births)
700
600
500
400
300
2000
2001
2002
2003
2004
2005
2006
2007
2008
2009
2010
2011
2012
2013
2014
2015
2016
2017
Year
IMD Quintiles (2015)
1 (Least deprived)
2
3
4
5 (Most deprived)

Supplementary Material: Child poverty and inequalities in infant mortality in England 2000-17: time trend analysis

Web appendix 1: Summary of dataset for local authority level analysis

Statistic	N	Mean	St. Dev.	Min	Max
Year	5,832	2,008	5	2,000	2,017
birth	5,832	1,975	1,605	222	17,766
death	5,832	9	11	0	169
imd_quintile	5,832	3	1	1	5
year_centred	5,832	8	5	0	17
year_knot13	5,832	1	1	0	4
imr	5,832	418	214	0	1,570

Web appendix 2: Jointpoint regression to identify breakpoint

We therefore investigated empirically whether there was a significant change in the trend in infant mortality between 2000 and 2017. We use an iterative search procedure to identify which breakpoint produced the best fitting mode by comparing all models with these alternative breakpoints at each year from 2001 to 2016, as well as models no breakpoints. We then plotted the Residual Mean Squared Error (RMSE) values from each of these models to identify the model that provided the best fit the data. In other words we fitted 16 separate models each with a different break points. The figure below shows the RMSE from each of these models – indicating that a breakpoint at 2013 provides the best fitting model compared to all the other alternative break points.

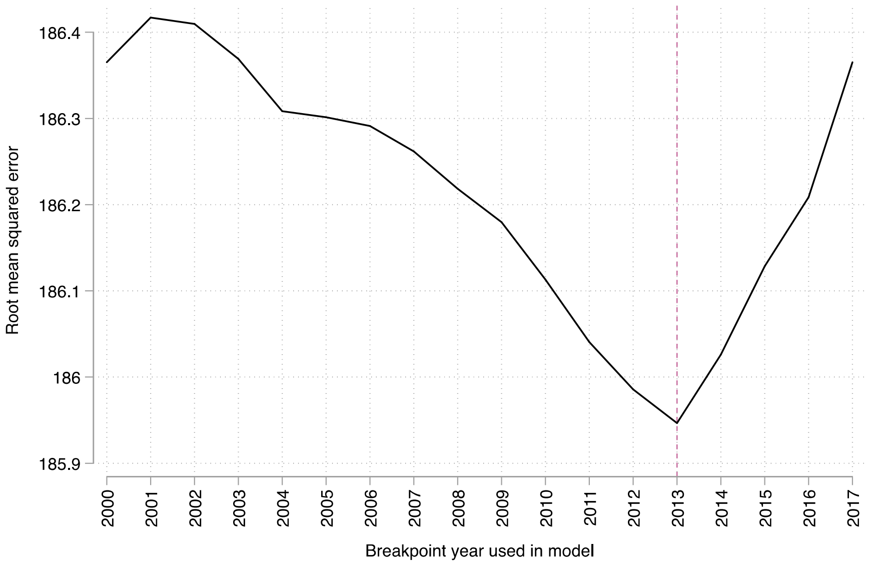

For peer review only - http://bmjopen.bmj.com/site/about/guidelines.xhtml

Web appendix 3: Segmented mixed effects regression model for infant mortality including two linear spline terms

We use a segmented mixed effects regression model for the mean response:

$$Y_{ij} = \beta_1 * \text{spline1} + \beta_2 * \text{spline2} + \beta_2 * \text{spline1} * \text{IMDQ} + \beta_4 * \text{spline2} * \text{IMDQ} + U_i + Z_{ij}$$

Let Y_{ij} is the *j*th repeated measurement (here, IMR) on the *i*th local area. Spline1 takes the value of year. Spline2 takes the value of *0* for year less than *2013*, taken to be the breakpoint for change in tren*d, and year − 2013* for year greater than *2013. This* defines a segmented regression with a change in slope of IMR at year=2013. IMDQ is IMD quintile, coded as a categorical variable. U_i is a random intercept term describing how the average IMR of the *i*th local area varies about the population-averaged response for all local areas with the same values of the explanatory variables. The model assumes that the U_i are independent copies of a Normally distributed random variable with mean zero and variance v^2. Z_{ij} is an error term that assumes that the Z_{ij} are independent copies of a Normally distributed random variable with mean zero and variance τ^2.

Web appendix 4: Linear mixed effects model results for trends in infant mortality at lower tier local authority level 2000-2017 – full R output with random effects.

	imr		
Predictors	*Estimates*	*CI*	*p*
(Intercept)	377.45	349.94 – 404.96	**<0.001**
year centred	-5.84	-8.70 – -2.98	**<0.001**
year knot 13	-4.19	-16.92 – 8.54	0.519
factor(imd quintile)2	66.13	27.38 – 104.89	**0.001**
factor(imd quintile)3	109.87	71.12 – 148.62	**<0.001**
factor(imd quintile)4	170.67	131.91 – 209.42	**<0.001**
factor(imd quintile)5	292.08	253.33 – 330.83	**<0.001**
year_centred:factor(imd_quintile)2	-2.67	-6.70 – 1.36	0.194
year_centred:factor(imd_quintile)3	-2.56	-6.59 – 1.47	0.213
year_centred:factor(imd_quintile)4	-7.27	-11.30 – -3.24	**<0.001**
year_centred:factor(imd_quintile)5	-12.43	-16.46 – -8.40	**<0.001**
year_knot13:factor(imd_quintile)2	15.65	-2.28 – 33.58	0.087
year_knot13:factor(imd_quintile)3	3.89	-14.04 – 21.83	0.671
year_knot13:factor(imd_quintile)4	20.50	2.57 – 38.43	**0.025**
year_knot13:factor(imd_quintile)5	24.14	6.20 – 42.07	**0.008**
**Random Effects**			
σ^2	34654.53		
$\tau_{00\ LA\_name}$	3953.12		
ICC_{LA_name}	0.10		
Observations	5832		
Marginal R^2 / Conditional R^2	0.159 / 0.245		

For peer review only - http://bmjopen.bmj.com/site/about/guidelines.xhtml

Web appendix 5: Residual diagnostics for local authority level analysis

The residuals from our longitudinal model plotted below. The residuals are normally distributed.

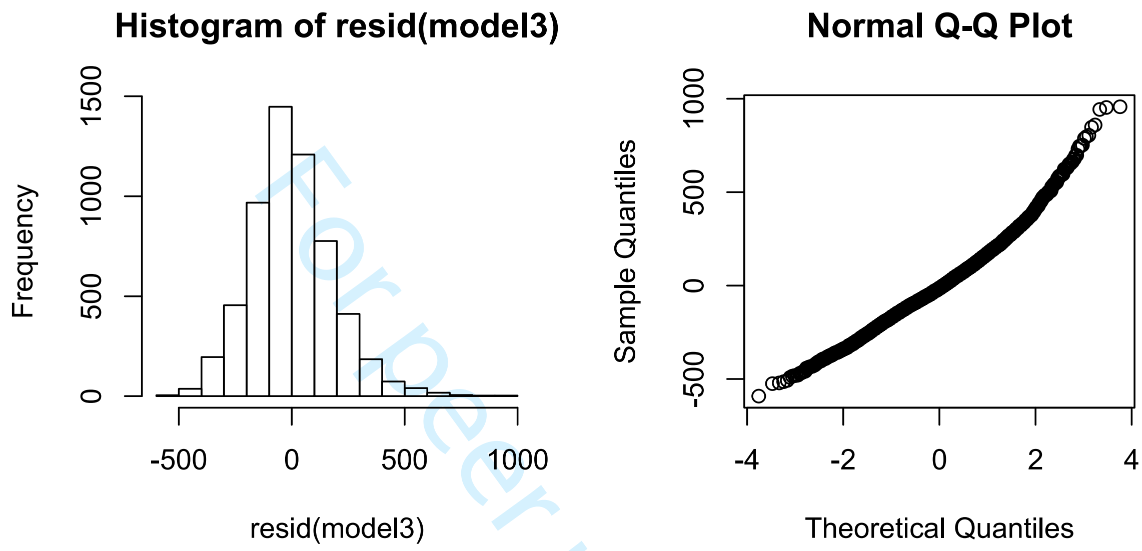

Web appendix 6: Modelling strategy for the fixed effects level analysis

Our cross-regional fixed effects model is as follows.

$Y_{ij} = \beta_1 \text{Child Poverty}_{ij} + \boldsymbol{\beta}_2 \text{Year} + \mu_i + Z_{ij}$

Let Y_{ij} is the infant mortality rate in the jth year in the ith English region,

Child Poverty$_{ij}$ is the child poverty rate in the jth year in the ith English region.

Year is a set of dummy variables for each year from 2000 to 2017

μ are regional fixed effects, and Z_{ij} are independent copies of a normally distributed random error term.

Web appendix 7: Residual diagnostics for regional fixed effects analysis

For peer review only

For peer review only - http://bmjopen.bmj.com/site/about/guidelines.xhtml

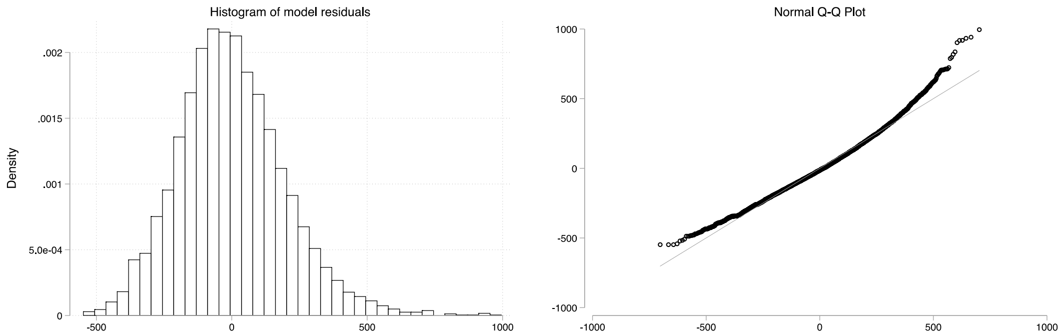

For peer review only

STROBE Statement—checklist of items that should be included in reports of observational studies

	Item No	Recommendation
**Title and abstract**	1	(*a*) Indicate the study's design with a commonly used term in the title or the abstract - Done : Title: **Child poverty and inequalities in infant mortality in England 2000-17: time trend analysis**
		(*b*) Provide in the abstract an informative and balanced summary of what was done and what was found<br>– Done – see ABSTRACT page 2
**Introduction**		
Background/rationale	2	Explain the scientific background and rationale for the investigation being reported. - Done see BACKGROUND page 4
Objectives	3	State specific objectives, including any prespecified hypotheses<br>Done see Abstract and BACKGROUND- "We therefore investigate the extent to which increases in child poverty may have led to increased infant mortality in recent years. Firstly, we investigate whether infant mortality increased more in those parts of the country with the greater numbers of people on a low income or receiving low income related welfare benefits. Secondly, we estimate whether there was a significant change in the trend in infant mortality in those areas and the timing of this change. Thirdly, we exploit regional differences in rates of infant mortality and child poverty to estimate the extent to which those regions with greater increases in child poverty experience greater increases in infant mortality. We use these estimates to calculate the proportion of recent infant mortality increases that are potentially explained by increases in child poverty."<br>Page 5
**Methods**		
Study design	4	Present key elements of study design early in the paper<br>– Done – see METHODS page 5
Setting	5	Describe the setting, locations, and relevant dates, including periods of recruitment, exposure, follow-up, and data collection. Done – see METHODS➔ Setting and data sources page 5
Participants	6	(*a*) *Cohort study*—Give the eligibility criteria, and the sources and methods of selection of participants. Describe methods of follow-up – NA – participants are the entire population<br>*Case-control study*—Give the eligibility criteria, and the sources and methods of case ascertainment and control selection. Give the rationale for the choice of cases and controls- NA<br>*Cross-sectional study*—Give the eligibility criteria, and the sources and methods of selection of participants - NA
		(*b*) *Cohort study*—For matched studies, give matching criteria and number of exposed and unexposed- NA<br>*Case-control study*—For matched studies, give matching criteria and the number of controls per case - NA

For peer review only

Variables	7	Clearly define all outcomes, exposures, predictors, potential confounders, and effect modifiers. Give diagnostic criteria, if applicable. Done see METHODS➔ Data sources page 5
Data sources/ measurement	8*	For each variable of interest, give sources of data and details of methods of assessment (measurement). Describe comparability of assessment methods if there is more than one group. Done see METHODS➔ Data sources page 5
Bias	9	Describe any efforts to address potential sources of bias- Done see METHODS➔ analysis page 6-7 – fixed effect approach
Study size	10	Explain how the study size was arrived at. NOTE: the study size is the entire population of England, analysed as aggregate data for 324 local authorities and 9 regions.
Quantitative variables	11	Explain how quantitative variables were handled in the analyses. If applicable, describe which groupings were chosen and why. Done see METHODS➔ analysis - page 5 – "Local authorities were grouped in 5 categories (quintiles) based on the income deprivation score of the 2015 Indices of Multiple Deprivation."
Statistical methods	12	(a) Describe all statistical methods, including those used to control for confounding. Done- Done see METHODS➔ analysis- page 6 & 7
		(*b*) Describe any methods used to examine subgroups and interactions Done see METHODS➔ analysis- page 6 &7 – "To do this we used the local authority panel data to estimate a segmented mixed effects regression model for infant mortality including two linear spline terms for time interacted with income deprivation quintile, with a breakpoint indicating the change in trend."
		(*c*) Explain how missing data were addressed- As the measures are based on the whole population this is not relevant. There was no missing data.
		(*d*) *Cohort study*—If applicable, explain how loss to follow-up was addressed - NA<br>*Case-control study*—If applicable, explain how matching of cases and controls was addressed- NA<br>*Cross-sectional study*—If applicable, describe analytical methods taking account of sampling strategy- NA
		(*e*) Describe any sensitivity analyses. Done see robustness tests – page 7. And Web appendix.

Continued on next page

For peer review only - http://bmjopen.bmj.com/site/about/guidelines.xhtml

**Results**		
Participants	13*	(a) Report numbers of individuals at each stage of study—eg numbers potentially eligible, examined for eligibility, confirmed eligible, included in the study, completing follow-up, and analysed- NA
		(b) Give reasons for non-participation at each stage- NA
		(c) Consider use of a flow diagram- NA
Descriptive data	14*	(a) Give characteristics of study participants (eg demographic, clinical, social) and information on exposures and potential confounders –NA
		(b) Indicate number of participants with missing data for each variable of interest- There were no missing data
		(c) *Cohort study*—Summarise follow-up time (eg, average and total amount)- NA
Outcome data	15*	*Cohort study*—Report numbers of outcome events or summary measures over time- NA
		Case-control study—Report numbers in each exposure category, or summary measures of exposure- NA
		Cross-sectional study—Report numbers of outcome events or summary measures- NA
Main results	16	(*a*) Give unadjusted estimates and, if applicable, confounder-adjusted estimates and their precision (eg, 95% confidence interval). Make clear which confounders were adjusted for and why they were included- Done: Unadjusted estimates -see Figure 1 and in web appendix 4, Adjusted estimates in table 1
		(*b*) Report category boundaries when continuous variables were categorized- NA
		(*c*) If relevant, consider translating estimates of relative risk into absolute risk for a meaningful time period- All results are given as both and relative absolute change
Other analyses	17	Report other analyses done—eg analyses of subgroups and interactions, and sensitivity analyses. Done see RESULTS ➔ robustness tests page 8 and web appendix 4.
**Discussion**		
Key results	18	Summarise key results with reference to study objectives. Done see discussion page 8
Limitations	19	Discuss limitations of the study, taking into account sources of potential bias or imprecision. Discuss both direction and magnitude of any potential bias- Done see discussion page 8
Interpretation	20	Give a cautious overall interpretation of results considering objectives, limitations, multiplicity of analyses, results from similar studies, and other relevant evidence- Done see discussion page 8-10
Generalisability	21	Discuss the generalisability (external validity) of the study results. Done see discussion page 10
**Other information**		
Funding	22	Give the source of funding and the role of the funders for the present study and, if applicable, for the original study on which the present article is based- Funding sources given page 11.

*Give information separately for cases and controls in case-control studies and, if applicable, for exposed and unexposed groups in cohort and cross-sectional studies.

Note: An Explanation and Elaboration article discusses each checklist item and gives methodological background and published examples of transparent reporting. The STROBE checklist is best used in conjunction with this article (freely available on the Web sites of PLoS Medicine at http://www.plosmedicine.org/, Annals of Internal Medicine at http://www.annals.org/, and Epidemiology at http://www.epidem.com/). Information on the STROBE Initiative is available at www.strobe-statement.org.

For peer review only - http://bmjopen.bmj.com/site/about/guidelines.xhtml

For peer review only

BMJ Open

Assessing the impact of rising child poverty on the unprecedented rise in infant mortality in England 2000-17: time trend analysis

Journal:	*BMJ Open*
Manuscript ID	bmjopen-2019-029424.R1
Article Type:	Research
Date Submitted by the Author:	23-May-2019
Complete List of Authors:	Taylor-Robinson, David; University of Liverpool, Department of Public Health and Policy<br>Lai, Eric; University of Liverpool, Public Health and Policy<br>Wickham, S; University of Liverpool<br>Rose, Tanith; University of Liverpool, Public Health and Policy<br>Norman, Paul; University of Leeds, School of Geography<br>Bambra, Clare; Newcastle University, Institute of Health & Society<br>Whitehead, Margaret; University of Liverpool, Public Health<br>Barr, Ben ; University of Liverpool, Department Public Health and Policy
**Primary Subject Heading**:	Public health
Secondary Subject Heading:	Epidemiology, Paediatrics, Health policy
Keywords:	EPIDEMIOLOGY, PUBLIC HEALTH, Community child health < PAEDIATRICS, Health policy < HEALTH SERVICES ADMINISTRATION & MANAGEMENT

SCHOLARONE™
Manuscripts

Assessing the impact of rising child poverty on the unprecedented rise in infant mortality in England 2000-17: time trend analysis

David Taylor-Robinson[1], Eric TC Lai[1], Sophie Wickham[1], Tanith Rose[1], Paul Norman[2], Clare Bambra[3], Margaret Whitehead[1], Ben Barr[1]

1. Department of Public Health and Policy, The Farr Institute@HeRC, Waterhouse Building 2nd Floor Block F, University of Liverpool, Liverpool, United Kingdom, L69 3GB.
2. School of Geography & Leeds Institute of Data Analytics, University of Leeds, Woodhouse Lane, Leeds, LS2 9JT
3. Institute of Health & Society, Baddiley-Clark, Richardson Road, Newcastle upon Tyne, NE2 4AX

Corresponding author: Professor David Taylor-Robinson; email: dctr@liv.ac.uk

For peer review only

For peer review only - http://bmjopen.bmj.com/site/about/guidelines.xhtml

Abstract

Objective

To determine whether there were inequalities in the sustained rise in infant mortality in England in recent years and the contribution of rising child poverty to these trends.

Design

Analysis of trends in infant mortality in local authorities grouped into 5 categories (quintiles) based on their level of income deprivation. Fixed effects regression models were used to quantify the association between regional changes in child poverty and regional changes in infant mortality.

Setting

324 English local authorities in 9 English Government Office Regions.

Participants

Live born children under 1 year of age.

Main outcome measure

Infant mortality rate (IMR), defined as the number of deaths in children under 1 year of age per 100,000 live births in the same year.

Results

The sustained and unprecedented rise in infant mortality in England from 2014-2017 was not experienced evenly across the population. In the most deprived local authorities, the previously declining trend in infant mortality reversed and mortality rose, leading to an additional 24 infant deaths per 100,000 live births per year (95% CI 6-42), relative to the previous trend. There was no significant change from the pre-existing trend in the most affluent local authorities. As a result, inequalities in infant mortality increased, with the gap between the most and the least deprived local authority areas widening by 52 deaths per 100,000 births (95% CI 36 to 68). Overall from 2014-2017, there were a total of 572 excess infant deaths (95% CI 200 to 944) compared to what would have been expected based on historical trends. We estimated that each 1% increase in child poverty was significantly associated with an extra 5.8 infant deaths per 100,000 live births (95%CI 2.4 to 9.2). The

For peer review only - http://bmjopen.bmj.com/site/about/guidelines.xhtml

findings suggest that about a third of the increases in infant mortality between 2014 and 2017 can be attributed to rising child poverty 172 deaths 95% CI (74 to 266).

Conclusion

This study provides evidence that the unprecedented rise in infant mortality disproportionately affected the poorest areas of the country, leaving the more affluent areas unaffected. Our analysis also linked the recent increase in infant mortality in England with rising child poverty, suggesting that about a third of the increase in infant mortality from 2014-17 may be attributed to rising child poverty.

For peer review only

For peer review only - http://bmjopen.bmj.com/site/about/guidelines.xhtml

Strengths and limitations of this study

- Use of national level data shows that the unprecedented rise in infant mortality in England has disproportionately affected the poorest areas of the country, leaving the more affluent areas unaffected
- Using linked area level data we show that the recent increase in levels of child poverty was associated with about a third of the extra infant deaths in England in the period 2014-17.
- Limitations include the lack of individual level data, and information on potentially mediating pathways, such as changes in health and social care spending on children that may have contributed.

Introduction

Infant mortality rate (IMR) has risen for the last four years in England, yet the role of increasing levels of child poverty in explaining these trends is unclear. Along with others[1], we raised concern about recent rises in infant mortality in England in two letters to the BMJ.[2,3] We noted that this rise had occurred particularly amongst more disadvantaged children from routine and manual socioeconomic groups. Rising infant mortality is unusual in high income countries, and international data show that infant mortality has continued to decline in most rich countries in recent years.[4] Infant mortality is a sensitive indicator of the changing overall health of societies and, as such, acts as an early warning system for future adverse trends. There is therefore an urgent need to understand this extremely concerning trend in England.

Adverse trends in mortality have occurred across all age groups in England in recent years.[5] Several commentators have suggested that these could be due to austerity policies introduced in recent years including cuts to NHS, local authority and public health services and changes to welfare benefits. [6,7] Since 2010, there have been sustained reductions in the welfare benefits available to families with children including: the abolition of child benefit and child tax credit for the third child or more; reductions in the value of tax credits, below inflation up-rating of most working-age benefits; housing benefit reforms including the under occupancy charge (most commonly referred to as 'bedroom tax'), introduction of universal credit; and household caps on total benefit receipt (regardless of how many children are in the household).[8] These welfare changes have disproportionately affected the most

For peer review only - http://bmjopen.bmj.com/site/about/guidelines.xhtml

For peer review only

deprived local authorities and regions [8] and have led to a rise in child poverty.[9] The impacts of these changes on trends in child health have not been considered. For example, a recent review of mortality increases by Public Health England did not consider the potential causes of increases in infant mortality.[5]

There is strong evidence that increased child poverty leads to deteriorating child health and increased infant deaths[10–14]. Whilst relative child poverty declined between 2007 and 2013, we are now seeing increases: child poverty (defined as living in a household with income below 60% of the median household after housing costs) rose by 2 percentage points between 2014 and 2017 and it is projected to increase further through to 2022.[9] By 2017 there were 4.1 million children in England living in relative poverty amounting to 30% of all English children. This compares to less than 10% of children in European countries such as Austria, Denmark, Finland, Iceland, Norway, Slovenia, Sweden, and Switzerland.[15] The recent rises in infant mortality in England have occurred concurrently with these increases in child poverty (figure 1).

Figure 1: Trends in infant mortality and child poverty in England, 2007-17

In this paper, we investigate whether there were inequalities in the sustained rise in infant mortality in England in recent years and the contribution of rising child poverty to these trends. Firstly, we investigate whether infant mortality increased more in those parts of the country with the highest numbers of people receiving low income related welfare benefits, as these are the areas that are most likely to have been adversely affected by recent changes in welfare policy. We estimate whether there was a significant change in the trend in infant mortality in those areas and the timing of this change. Secondly, we exploit regional differences in trends in infant mortality and child poverty to estimate the extent to which those regions with greater increases in child poverty experienced greater increases in infant mortality. We use these estimates to calculate the proportion of recent infant mortality increases that are potentially explained by increases in child poverty.

For peer review only - http://bmjopen.bmj.com/site/about/guidelines.xhtml

Methods

Annual Vital Statistics data for the number of registered infant deaths (< 1 year of age) and live births for 324 lower tier local authority level (324 local areas) between 2000 to 2017 were obtained from the Office of National Statistics. We chose this period due to data availability and since it captures a contemporary period during which both infant mortality and child poverty rates have changed dramatically. Local authorities were grouped in 5 categories (quintiles) based on the income deprivation score of the 2015 Indices of Multiple Deprivation. This score is a non-overlapping count of the numbers of people in each local authority on a low income and in receipt of means tested benefits as a proportion of the population.[16]

Regional relative child poverty was measured as the proportion of children living in households in each region with less than 60 per cent of contemporary national median household income, using the Households Below Average Income data provided by the Department for Work and Pensions.[17] This is the most widely used measure of poverty within the European Union and recognises that the experience of poverty is relative to standards of living that are considered normal within a society and that these average standards change over time. [14] As this measure not available at the local authority level we used data for the nine government office regions of England. [18] See web appendix 1 for a summary of the dataset.

Patient and Public Involvement

Our study was informed by discussions with children and young people's reference groups in Liverpool, who encouraged us to undertake analyses focussed on developing a better understanding of social factors that shape children and young people's (CYP) lives, such as child poverty. The results of our work are feeding into ongoing discussion with CYP about health inequalities, informing the UNICEF Child Friendly City programme in Liverpool.

Statistical analysis

The statistical analysis proceeded in three steps. Firstly, we assessed descriptive trends in infant mortality between 2000 and 2017 for the five groups of local authorities categorised by

For peer review only - http://bmjopen.bmj.com/site/about/guidelines.xhtml

their level of income deprivation (see above). Secondly, we tested whether there was a statistically significant change in the infant mortality trend during this time, the timing of that change in trend and whether any change in trend differed by the level of income deprivation. To do this we used longitudinal local authority area level data to estimate a segmented mixed effects regression model for infant mortality including two linear spline terms for time interacted with income deprivation quintile, with a breakpoint indicating the change in trend. We used an iterative search procedure to identify which breakpoint provided the best fit for the data, [19,20] in other words identifying at which time point a change in trend occurred. This model included random effects (intercepts) at the local authority level to take account of the longitudinal nature of the data (see appendix for details). Secondly, we used this model to estimate whether there had been a significant change in trend in recent years, whether this had led to widening inequalities between local authority areas based on their level of income deprivation and to estimate the level of excess mortality that was attributable to any change in trend. Excess mortality was estimated as the marginal difference between the observed trend and that which would be predicted from the model if pre-existing trends had continued, using the *margins[21]* commands in Stata version 14

In the third step of the analysis, we assessed the association of changes in regional child poverty and regional infant mortality. As there is potential confounding from unobserved factors that vary between regions, or national trends in unobserved factors that affect all region, we used a fixed effects approach to remove between region differences and national trends.[22,23] This conservative approach involves including dummy variables for each region and year to assess the association between deviations from the average rate of change in poverty and deviations from average rate of change in infant mortality in each region (see web appendix 2-3 for further details). This method means that the estimated association between child poverty and infant mortality cannot be confounded by any time invariant differences between regions or any national trend that effects all regions, providing an estimate that is likely to reflect a causal association. We aligned annual regional child poverty rates for financial years to infant mortality registered in the calendar year in which the financial year ended. In other words, 2016-17 child poverty data was aligned with 2017 infant mortality data, meaning that child poverty measures were lagged by 9 months compared to infant mortality measures. We used robust clustered standard errors to ensure that they are robust to serial correlation in the data. This model was used to estimate the number of child deaths attributable to child poverty increases in recent years, by estimating

For peer review only - http://bmjopen.bmj.com/site/about/guidelines.xhtml

the marginal difference between the observed trend and that which would be predicted from the model if child poverty had not increased *[21]*. All models were estimated using Stata version 14 and R version 3.5.1. As a robustness test we repeated our analysis stratified by neonatal and post-neonatal deaths.

Results

Infant mortality had been falling for all groups of local authority areas since 2000, with greater decreases in the most income deprived areas, reducing inequalities. From 2013, this trend changed, and infant mortality increased particularly in the most income deprived local authority areas (figure 2). In web appendix 4 we have provided a link to a visualisation of the geographical distribution of the IMR data for England.

Figure 2: Infant mortality trend by deprivation quintile of local authority district, 2000-2017, with 95% binomial confidence intervals.

The breakpoint analysis also showed a significant change in trend from 2013 (appendix). Table 1 shows the results of the regression model for trends in infant mortality since that time. From 2013-17 there was a dramatic reversal of the pre-existing trend, with an additional annual increase of 24 deaths per 100,000 live births (95%CI 6 to 42) in the most deprived quintile 5 compared to the 2000-2013 trend. A significant change in trend was also observed for quintile 4, whilst there was no significant change from the previously declining trend in the most affluent quintile. The gap in infant mortality between the most income deprived and most affluent quintile increased by 52 deaths per 100,000 births (95% CI 36 to 68). Based upon these trends we estimated that there were an additional 570 excess deaths (95% CI 200 to 944) in the period 2014-17 than would have been expected if the historical trends had continued.

Table 1: Segmented regression model results for trends in infant mortality at lower tier local authority level 2014-2017 (per 100,000 live births per year)

Level of income deprivation	Annual change (deaths per 100,000) in infant mortality 2014-2017 relative to previous trend (2000-2013) (95% CI)	P value for change in trend from previous period

For peer review only - http://bmjopen.bmj.com/site/about/guidelines.xhtml

Quintile 1 (most affluent)	-4.19	[-16.91,8.53]	0.518
Quintile 2	15.65	[-2.27,33.57]	0.087
Quintile 3	3.89	[-14.03,21.81]	0.67
Quintile 4	20.5	[2.58,38.42]	0.025
Quintile 5 (most deprived)	24.14	[6.22,42.05]	0.008

Estimates based on random effects regression model using LA panel dataset of infant mortality from 2000 to 2017, N= 5,832 local authority years. See web appendix 5-9 for full model output and checks.

In the fixed effects analysis each percentage point change in child poverty was associated with a change in infant mortality of 5.8 deaths per 100,000 live births (95% confidence interval 2.4 to 8.9). From this model we estimate that 172 deaths 95% CI (74 to 266) between 2014 and 2017 were attributable to increases in child poverty, almost a third of the overall rise in infant mortality over that period. Repeating our analysis for neonatal and post-neonatal deaths showed similar results, with a significant change in trend in the most disadvantaged quintile for both outcomes, though the change was most pronounced for neonatal deaths (see web appendix 10).

Discussion

Since 2013, infant mortality has increased in England and there have been an additional 570 infant deaths over 4 years (2014 to 2017) compared to what would have been expected based on recent historical trends. These excess deaths have largely occurred in the most disadvantaged areas, increasing inequalities. Our study estimated that the recent increase in levels of child poverty was associated with 172 (about a third) of the extra infant deaths in England in the period 2014-17.

Limitations of the study

Before evaluating the implications of our findings, we note several important limitations. Firstly, due to absence of individual level data, we undertook an observational analysis at the population level. Lack of data on levels of child poverty at a small area level meant that we undertook our analysis of the effects of child poverty using child poverty estimates for 9 regions within England. This meant we could take advantage of regional differences in trends over time, and use a fixed effects approach to adjust for time invariant confounding variables.

For peer review only - http://bmjopen.bmj.com/site/about/guidelines.xhtml

We were not able, however, to estimate the effect of increases in child poverty on trends in infant mortality for groups of local authorities within these regions. As our main analysis was based on aggregate data, however, we cannot identify whether the additional infant deaths were in the same families who have experienced a rise in child poverty. It is also possible that the association between the child poverty and infant mortality in our analysis was due to trends in unobserved time-varying confounding factors that varied between regions.

Secondly, we measured child poverty using the relative poverty measure used by the Department for Work and Pensions in England, because it is used internationally, as well as in the UK. Furthermore, whether or not relative measures of income poverty effectively reflect children's life chances has been the focus of policy debates in the UK.[24] This measure, however, is a simplification of underlying income trajectories whereby families with children move in and out of poverty over time. Furthermore, the measure we have used is likely to underestimate the extent of the experience of poverty in England. A recent report published by the Social Metrics Commission outlining a new measure of poverty which extends beyond income-based poverty, suggests that there are 4.5 million children living (32.6%) in poverty in the UK.[25] In addition, the Joseph Rowntree Foundation's (JRF) has shown that the proportion of children living below the Minimum Income Standard has increased from 39% to 45% (an increase of about 1 million) between 2008-09 and 2014-15.[26] Further studies could usefully explore the relationship between a continuous measure of income and aspects of child health using individual level data, in order to identify any policy relevant threshold effects.

Thirdly, we did not have data on cause of death, and were not able to investigate the factors mediating the association between rising child poverty and rising infant mortality, such as birth characteristics (for example preterm birth, maternal age and maternal smoking) and post-natal factors (for example breastfeeding and child care in the first year of life).[27,28] Fourthly, it is important that further research investigates the potential role of parental characteristics, gestational age and other risk factors for child mortality in explaining the observed trends, in addition to assessing how changes in health and social care spending on children may have contributed. [6,7]

For peer review only - http://bmjopen.bmj.com/site/about/guidelines.xhtml

Comparison with previous studies

To our knowledge this is the first study to explore both the inequalities in the unprecedented rise in infant mortality in England on the basis of area deprivation and the reasons behind this rise. Whilst the UK government recently commissioned a report into adverse mortality trends, infant mortality was not considered in any detail. [5] Our analysis corroborates our previous analysis of trends in infant mortality on the basis of parental occupational social class, which suggested that IMR was rising particularly in the most disadvantaged social groups.[3,29] A recent report by the Royal College of Paediatrics and Child Health (RCPCH) also noted the reversal of over 100 year of declining infant mortality in England and Wales, and that rises in infant mortality were most striking in the deprived portion of the population. [27] The RCPCH further assesses various scenarios of infant mortality, for instance showing that even if infant mortality begins to decline again at its previous rate, rates will be 80% higher than the EU15+ average in 2030. Our study uses the most up-to date infant mortality data, and suggests that rather than just stalling, infant mortality is rising sharply in the most disadvantaged areas.

In addition to increasing inequalities in infant mortality, social inequalities in numerous aspects of health in England appear to be rising.[7,19,30,31] Barr and colleagues demonstrated a reversal in trends in inequalities in life expectancy.[19] Two analyses from the Global Burden of Diseases collaboration have shown both a stagnation in the improvement in life expectancy in England; and increasing inequalities since 2010.[30,31] Furthermore Bennett et al identify deaths in children younger than 5 years as a major contributor to inequality in life expectancy.[30] A further analysis has demonstrated an increase in the North-South divide in health in England.[7]

Our study shows that rising income poverty may be contributing to rising infant mortality. This finding is likely to be generalisable to other high income settings, since numerous studies conducted in Western Europe and the USA have shown an association between social disadvantage and infant mortality. [32–35] Furthermore, numerous studies have suggested that there is a causal link between increasing child poverty and a deterioration in various aspects of child health and development [10–14] For example, a recent systematic review by Cooper and Stewart concluded that "poorer children have worse cognitive, social-behavioural and health

For peer review only - http://bmjopen.bmj.com/site/about/guidelines.xhtml

outcomes in part because they are poorer, and not just because poverty is correlated with other household and parental characteristics." Their review demonstrated clear causal links between income poverty and a range of child health outcomes, including only studies that applied quasi-experimental designs.[13,36] Komro and colleagues, for example, used a difference-in-differences research design to assess the impact of state-level minimum wage in the USA on infant mortality. The authors show that each dollar increase in the minimum wage above the federal level was associated with a 1% to 2% decrease in low birth weight births and a 4% decrease in postneonatal mortality. [37] Additionally, the results of a longitudinal analysis of social welfare expenditure data from 19 Organisation for Economic Co-operation and Development (OECD) countries over the time period from 1980 to 2010, showed that cash benefits for families have positive effects on reducing infant mortality, with a $10 increase in family cash allowances per child predicting a reduction in infant mortality by 4% ($p = .007$).[12] Previous research has shown that expansions to social security nets and public services have decreased inequalities in life expectancy [19], infant mortality rates [38], and mortality amenable to healthcare.[39] Robinson et al, for example, found that the English health inequalities strategy (2000-2010) was associated with decreases in inequalities in infant mortality in England. [38] Similarly, Krieger et al found that the 1960s 'War on Poverty' led to decreases in inequalities in IMR in the USA.[40]

For peer review only - http://bmjopen.bmj.com/site/about/guidelines.xhtml

Policy implications

Our analysis has important implications in the context of the projected ongoing increase in levels of child poverty in the UK. For the first time in nearly 40 years there has been a sustained increase in infant mortality in the poorest areas. English regions with the largest rises in child poverty have had the largest increases in infant mortality. Our analysis suggests that the weakened social protection safety net - leading to rising levels of child poverty – may be contributing to this rise in mortality in the most disadvantaged infants. Our analysis suggests that the rise in child poverty explains approximately a third of this rise. This could be an underestimate of the impact if the measures of child poverty used are an underestimate of the true increase in disadvantage facing children (see above). It may also be the case that other policy changes occurring at this time such as the real terms cuts to NHS, local authority children's services, social care and public health budgets –are also be contributing to the rise in mortality as has been suggested elsewhere. [6,7]

Infant mortality is an uncommon event and represents the most severe tip of the iceberg in terms of the impact of social conditions on health. It can, however, act as an early warning system or litmus test of the overall health of societies. Rising poverty is likely to be having a myriad of adverse impacts on other aspects of child health that will have repercussions across the lifecourse.[36] In the context of increasing health inequalities in England, policies that reduce poverty and social inequalities, investing in child health and social care are likely to reduce the occurrence of infant mortality and that of many other adverse child health outcomes.[30]

This rise in mortality in the most disadvantaged children is unprecedented and requires urgent action by national and local governments and the health and social care system. It is likely that the rise in child poverty is an important factor contributing to this trend. As the United Nations (UN) has recently highlighted, rising poverty in the UK is a political choice[41] and it is time for the government to reverse this trend establishing a welfare system that protects children from poverty.

What is already known on this topic

For peer review only - http://bmjopen.bmj.com/site/about/guidelines.xhtml

In 2013, infant mortality rates began to rise in England, reversing a long-term decline. It is not clear whether this increase affected all sections of the population evenly and how much can be attributed to rising levels of child poverty.

What this study adds

The sustained increase in infant mortality in England from 2013-17 has occurred in the most disadvantaged areas, while more affluent areas continued in their previous declining trend. This rise in infant mortality in disadvantaged areas is partially explained by rising child poverty.

For peer review only

For peer review only - http://bmjopen.bmj.com/site/about/guidelines.xhtml

Funding: DTR and EL are funded by the MRC on a Clinician Scientist Fellowship (MR/P008577/1). SW is supported by a Wellcome Trust Society and Ethics fellowship (grant number 200335/Z/15/Z). BB and TR are supported by the National Institute for Health Research (NIHR) Collaboration for Leadership in Health Research and Care (CLAHRC NWC) and the NIHR School for Public Health Research.

The MRC, Wellcome Trust and NIHR had no role in the study design, data collection and analysis, decision to publish, or preparation of the manuscript. This report is independent research arising from research supported by the MRC, Wellcome Trust and NIHR. The views expressed in this publication are those of the authors and not necessarily those of the NHS, the NIHR, or the Department of Health and Social Care.

Competing interest statement

All authors have completed the ICMJE uniform disclosure format www.icmje.org/coi_disclosure.pdf and declare: DTR and EL are funded by the MRC on a Clinician Scientist Fellowship (MR/P008577/1). SW is supported by a Wellcome Trust Society and Ethics fellowship (grant number 200335/Z/15/Z). BB and TR are supported by the National Institute for Health Research (NIHR) Collaboration for Leadership in Health Research and Care (CLAHRC NWC); no financial relationships with any organisations that might have an interest in the submitted work in the previous three years; no other relationships or activities that could appear to have influenced the submitted work.

Contributorship statement

DTR is lead author and guarantor. DTR and BB planned the study and led the drafting and revising of the manuscript. DTR, BB and EL conducted the analysis. DTR, BB, EL, SW, TR, PN, CB and MMW contributed to interpretation of the data, drafting of the manuscript, and revisions. All authors agreed the submitted version of the manuscript.

Transparency declaration

DTR is the guarantor and affirms that the manuscript is an honest, accurate, and transparent account of the study being reported; that no important aspects of the study have been omitted;

For peer review only - http://bmjopen.bmj.com/site/about/guidelines.xhtml

For peer review only

and that any discrepancies from the study as planned (and, if relevant, registered) have been explained.

Ethics committee approval

None required

Data sharing

Statistical code available on request from lead author

Licence statement

The Corresponding Author has the right to grant on behalf of all authors and does grant on behalf of all authors, a worldwide licence (http://www.bmj.com/sites/default/files/BMJ%20Author%20Licence%20March%202013.doc) to the Publishers and its licensees in perpetuity, in all forms, formats and media (whether known now or created in the future), to i) publish, reproduce, distribute, display and store the Contribution, ii) translate the Contribution into other languages, create adaptations, reprints, include within collections and create summaries, extracts and/or, abstracts of the Contribution and convert or allow conversion into any format including without limitation audio, iii) create any other derivative work(s) based in whole or part on the on the Contribution, iv) to exploit all subsidiary rights to exploit all subsidiary rights that currently exist or as may exist in the future in the Contribution, v) the inclusion of electronic links from the Contribution to third party material where-ever it may be located; and, vi) licence any third party to do any or all of the above. All research articles will be made available on an open access basis (with authors being asked to pay an open access fee—see http://www.bmj.com/about-bmj/resources-authors/forms-policies-and-checklists/copyright-open-access-and-permission-reuse). The terms of such open access shall be governed by a Creative Commons licence—details as to which Creative Commons licence will apply to the research article are set out in our worldwide licence referred to above.

For peer review only

For peer review only - http://bmjopen.bmj.com/site/about/guidelines.xhtml

References

1 Hiam L, Dorling D. Government's misplaced prevention agenda. *BMJ* 2018;**363**:k5134. doi:10.1136/bmj.k5134

2 Taylor-Robinson D, Barr B. Death rate now rising in UK's poorest infants. *BMJ* 2017;**357**:j2258. doi:10.1136/bmj.j2258

3 Death rate continues to rise for poorest infants in England and Wales. *The BMJ* Published Online First: 13 September 2018.https://www.bmj.com/content/360/bmj.k1090/rr-2 (accessed 17 Sep 2018).

4 Health status - Infant mortality rates - OECD Data. theOECD. http://data.oecd.org/healthstat/infant-mortality-rates.htm (accessed 23 Nov 2018).

5 Public Health England. A review of recent trends in mortality in England. London: : PHE 2018.

6 Bennett JE, Li G, Foreman K, *et al.* The future of life expectancy and life expectancy inequalities in England and Wales: Bayesian spatiotemporal forecasting. *The Lancet* 2015;**386**:163–70. doi:10.1016/S0140-6736(15)60296-3

7 Kontopantelis E, Buchan I, Webb RT, *et al.* Disparities in mortality among 25–44-year-olds in England: a longitudinal, population-based study. *The Lancet Public Health* 2018;**0**. doi:10.1016/S2468-2667(18)30177-4

8 Beatty C, Fothergill S. The uneven impact of welfare reform : the financial losses to places and people. 2016. doi:10.7190/cresr.2017.5563239352

9 Hood A, Waters T, Institute for Fiscal Studies (Great Britain), *et al. Living standards, poverty and inequality in the UK: 2016-17 to 2021-22.* 2017.

10 Wang H, Liddell CA, Coates MM, *et al.* Global, regional, and national levels of neonatal, infant, and under-5 mortality during 1990–2013: a systematic analysis for the Global Burden of Disease Study 2013. *The Lancet* 2014;**384**:957–79. doi:10.1016/S0140-6736(14)60497-9

11 Zylbersztejn A, Gilbert R, Hjern A, *et al.* Child mortality in England compared with Sweden: a birth cohort study. *The Lancet* 2018;**0**. doi:10.1016/S0140-6736(18)30670-6

12 Shim J. Social Welfare Expenditures and Infant Mortality. *Soc Work Public Health* 2015;**30**:567–77. doi:10.1080/19371918.2015.1073648

13 Cooper K, Stewart K. *Does Money Affect Children's Outcomes?: A Systematic Review.* Joseph Rowntree Foundation 2013.

14 Wickham S, Anwar E, Barr B, *et al.* Poverty and child health in the UK: using evidence for action. *Archives of disease in childhood* Published Online First: 2016. doi:10.1136/archdischild-2014-306746

15 OECD. Child_Poverty. https://www.oecd.org/els/CO_2_2_Child_Poverty.pdf (accessed 17 Dec 2018).

For peer review only - http://bmjopen.bmj.com/site/about/guidelines.xhtml

16 English indices of deprivation 2015. GOV.UK. https://www.gov.uk/government/statistics/english-indices-of-deprivation-2015 (accessed 10 Dec 2018).

17 Households below average income (HBAI) statistics. GOV.UK. https://www.gov.uk/government/collections/households-below-average-income-hbai--2 (accessed 23 Nov 2018).

18 ONS. Administrative geography. https://www.ons.gov.uk/methodology/geography/ukgeographies/administrativegeography/england (accessed 14 Dec 2018).

19 Barr B, Higgerson J, Whitehead M. Investigating the impact of the English health inequalities strategy: time trend analysis. *BMJ* 2017;**358**:j3310. doi:10.1136/bmj.j3310

20 Crawley M. *An Introduction to Statistics using R*. Chichechester: : John Wiley and Sons 2005.

21 Marginal analysis | Stata. https://www.stata.com/features/overview/marginal-analysis/ (accessed 18 Dec 2018).

22 Barr B, Taylor-Robinson D, Scott-Samuel A, *et al.* Suicides associated with the 2008-10 economic recession in England: time trend analysis. *BMJ* 2012;**345**:e5142. doi:10.1136/bmj.e5142

23 Barr B, Taylor-Robinson D, Stuckler D, *et al.* 'First, do no harm': are disability assessments associated with adverse trends in mental health? A longitudinal ecological study. *J Epidemiol Community Health* 2015;:jech-2015-206209. doi:10.1136/jech-2015-206209

24 FPH. APPG Inquiry: Child Poverty and Health. https://www.fph.org.uk/media/1374/appg-health-in-all-policies-inquiry-into-child-poverty-and-health2.pdf (accessed 14 Dec 2018).

25 Stroud P. The final report of the Social Metrics Commission: A new measure of poverty for the UK. https://lif.blob.core.windows.net/lif/docs/default-source/default-library/legj6470-measuring-poverty-full_report-181004-web.pdf?sfvrsn=0

26 Households below a Minimum Income Standard: 2008/09 to 2014/15. JRF. 2017.https://www.jrf.org.uk/report/households-below-minimum-income-standard-200809-201415 (accessed 10 Dec 2018).

27 Child health in England in 2030: comparisons with other wealthy countries | RCPCH. https://www.rcpch.ac.uk/resources/child-health-england-2030-comparisons-other-wealthy-countries (accessed 10 Dec 2018).

28 Sidebotham P, Fraser J, Covington T, *et al.* Understanding why children die in high-income countries. *Lancet* 2014;**384**:915–27. doi:10.1016/S0140-6736(14)60581-X

29 Taylor-Robinson D, Barr B. Death rate now rising in UK's poorest infants. *BMJ* 2017;**357**:j2258. doi:10.1136/bmj.j2258

For peer review only - http://bmjopen.bmj.com/site/about/guidelines.xhtml

30 Bennett JE, Pearson-Stuttard J, Kontis V, *et al.* Contributions of diseases and injuries to widening life expectancy inequalities in England from 2001 to 2016: a population-based analysis of vital registration data. *The Lancet Public Health* 2018;**3**:e586–97. doi:10.1016/S2468-2667(18)30214-7

31 Steel N, Ford JA, Newton JN, *et al.* Changes in health in the countries of the UK and 150 English Local Authority areas 1990–2016: a systematic analysis for the Global Burden of Disease Study 2016. *The Lancet* 2018;**392**:1647–61. doi:10.1016/S0140-6736(18)32207-4

32 Kim D, Saada A. The social determinants of infant mortality and birth outcomes in Western developed nations: a cross-country systematic review. *Int J Environ Res Public Health* 2013;**10**:2296–335. doi:10.3390/ijerph10062296

33 Weightman AL, Morgan HE, Shepherd MA, *et al.* Social inequality and infant health in the UK: systematic review and meta-analyses. *BMJ Open* 2012;**2**:e000964. doi:10.1136/bmjopen-2012-000964

34 Gisselmann MD. The influence of maternal childhood and adulthood social class on the health of the infant. *Soc Sci Med* 2006;**63**:1023–33. doi:10.1016/j.socscimed.2006.03.015

35 Kihal-Talantikite W, Padilla CM, Lalloué B, *et al.* Green space, social inequalities and neonatal mortality in France. *BMC Pregnancy Childbirth* 2013;**13**:191. doi:10.1186/1471-2393-13-191

36 Cooper K, Stewart K. Money Matters: Does Money Affect Children's Outcomes? A Systematic Review Update. http://sticerd.lse.ac.uk/case/_new/research/money_matters/default.asp

37 Komro KA, Livingston MD, Markowitz S, *et al.* The Effect of an Increased Minimum Wage on Infant Mortality and Birth Weight. *Am J Public Health* 2016;**106**:1514–6. doi:10.2105/AJPH.2016.303268

38 Robinson TJ, Barr B, Brown HW, *et al.* OP18 Evaluating the impact of the english health inequalities strategy on socioeconomic inequalities in the regional infant mortality rate. *J Epidemiol Community Health* 2018;**72**:A9–A9. doi:10.1136/jech-2018-SSMabstracts.18

39 Barr B, Bambra C, Whitehead M. The impact of NHS resource allocation policy on health inequalities in England 2001-11: longitudinal ecological study. *BMJ* 2014;**348**:g3231. doi:10.1136/bmj.g3231

40 Krieger N, Rehkopf DH, Chen JT, *et al.* The fall and rise of US inequities in premature mortality: 1960-2002. *PLoS Med* 2008;**5**:e46. doi:10.1371/journal.pmed.0050046

41 Poverty is a political choice. *The Lancet Public Health* 2018;**3**:e555. doi:10.1016/S2468-2667(18)30243-3

For peer review only

For peer review only - http://bmjopen.bmj.com/site/about/guidelines.xhtml

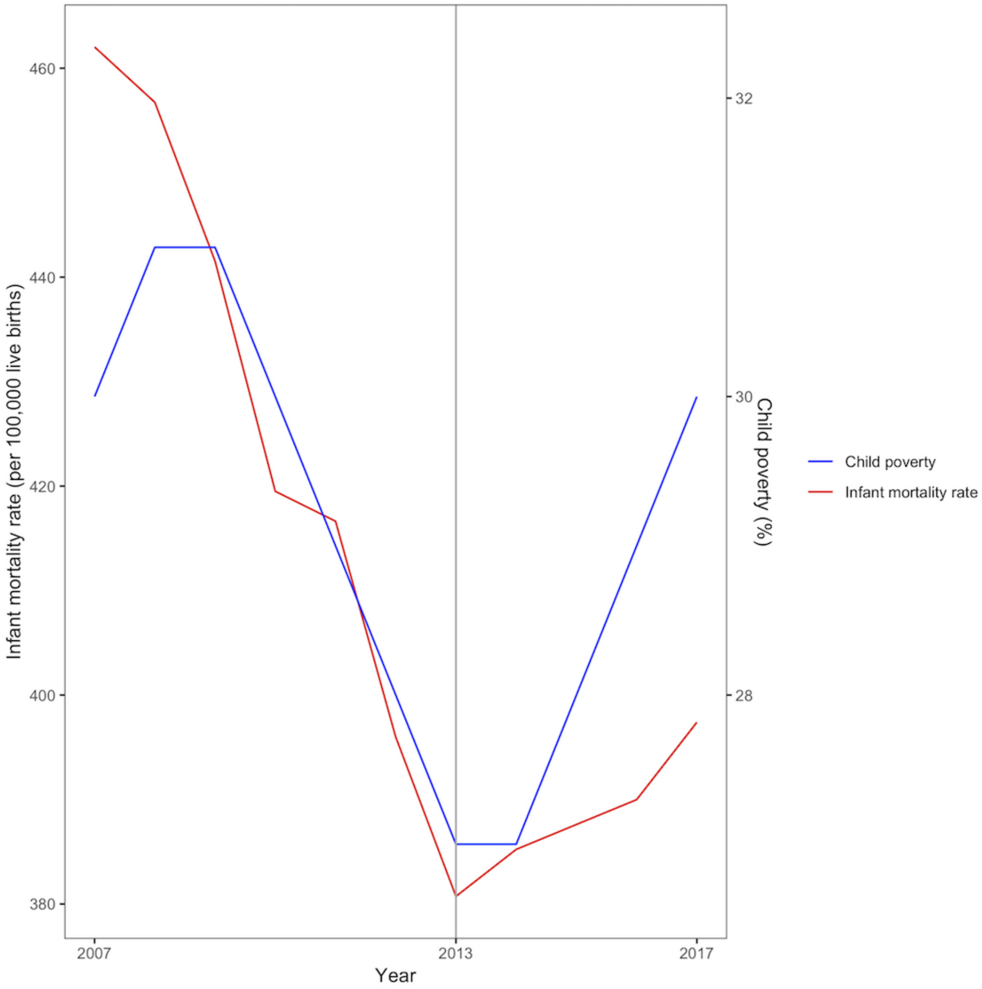

90x90mm (300 x 300 DPI)

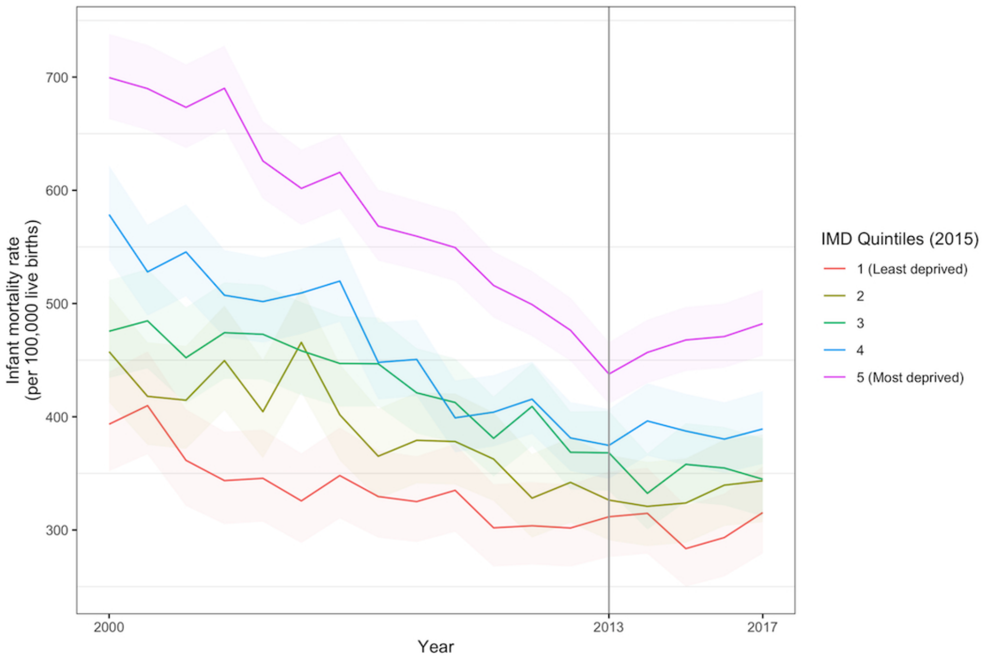

90x60mm (300 x 300 DPI)

Supplementary Material: Child poverty and inequalities in infant mortality in England 2000-17: time trend analysis

Web appendix 1: Summary of dataset for local authority level analysis

Statistic	N	Mean	St. Dev.	Min	Max
Year	5,832	2,008	5	2,000	2,017
birth	5,832	1,975	1,605	222	17,766
death	5,832	9	11	0	169
imd_quintile	5,832	3	1	1	5
year_centred	5,832	8	5	0	17
year_knot13	5,832	1	1	0	4
imr	5,832	418	214	0	1,570

Web appendix 2: Jointpoint regression to identify breakpoint

We therefore investigated empirically whether there was a significant change in the trend in infant mortality between 2000 and 2017. We use an iterative search procedure to identify which breakpoint produced the best fitting mode by comparing all models with these alternative breakpoints at each year from 2001 to 2016, as well as models no breakpoints. We then plotted the Residual Mean Squared Error (RMSE) values from each of these models to identify the model that provided the best fit with the data. In other words we fitted 16 separate models each with a different break points. The figure below shows the RMSE from each of these models – indicating that a breakpoint at 2013 provides the best fitting model compared to all the other alternative break points.

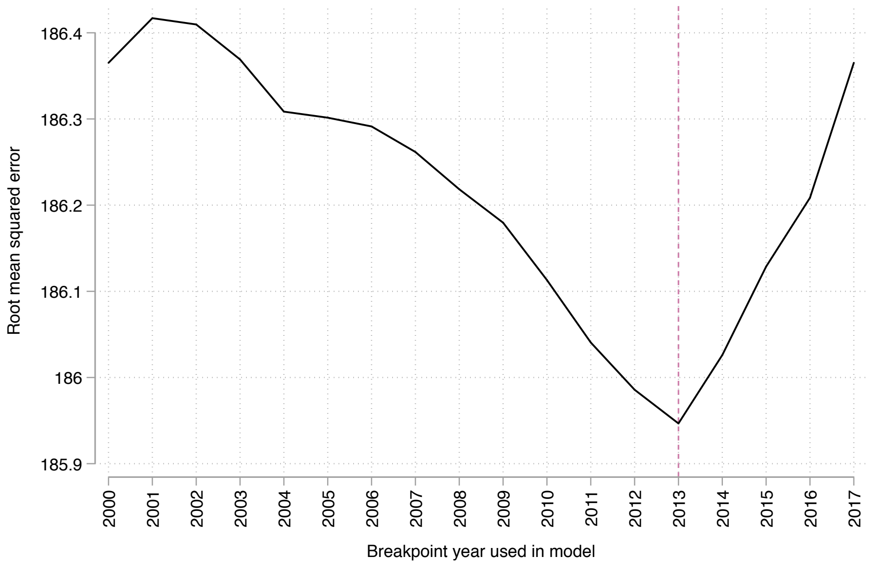

For peer review only - http://bmjopen.bmj.com/site/about/guidelines.xhtml

Web appendix 3: Segmented mixed effects regression model for infant mortality including two linear spline terms

We use a segmented mixed effects regression model for the mean response:

$$Y_{ij} = \beta_1\text{* spline1+ }\beta_2\text{*spline2 + }\beta_2\text{*spline1*IMDQ+ }\beta_4\text{*spline2*IMDQ + }U_i + Z_{ij}$$

Let Y_{ij} is the *j*th repeated measurement (here, IMR) on the *i*th local area. Spline1 takes the value of year. Spline2 takes the value of *0* for year less than *2013*, taken to be the breakpoint for change in tren*d, and year* − *2013* for year greater than *2013. This* defines a segmented regression with a change in slope of IMR at year=2013. IMDQ is IMD quintile, coded as a categorical variable. U_i is a random intercept term describing how the average IMR of the *i*th local area varies about the population-averaged response for all local areas with the same values of the explanatory variables. The model assumes that the U_i are independent copies of a Normally distributed random variable with mean zero and variance v^2. Z_{ij} is an error term that assumes that the Z_{ij} are independent copies of a Normally distributed random variable with mean zero and variance τ^2.

Web appendix 4: Link showing show the distribution of infant mortality across England
https://dblalex.carto.com/builder/c81de0d5-f893-4109-9471-b4b55afa351e/embed

Web appendix 5: Linear mixed effects model results for trends in infant mortality at lower tier local authority level 2000-2017 – full R output with random effects.

	**imr**		
Predictors	*Estimates*	*CI*	*p*
(Intercept)	377.45	349.94 – 404.96	**<0.001**
year centred	-5.84	-8.70 – -2.98	**<0.001**
year knot 13	-4.19	-16.92 – 8.54	0.519
factor(imd quintile)2	66.13	27.38 – 104.89	**0.001**
factor(imd quintile)3	109.87	71.12 – 148.62	**<0.001**
factor(imd quintile)4	170.67	131.91 – 209.42	**<0.001**
factor(imd quintile)5	292.08	253.33 – 330.83	**<0.001**
year_centred:factor(imd_quintile)2	-2.67	-6.70 – 1.36	0.194
year_centred:factor(imd_quintile)3	-2.56	-6.59 – 1.47	0.213
year_centred:factor(imd_quintile)4	-7.27	-11.30 – -3.24	**<0.001**
year_centred:factor(imd_quintile)5	-12.43	-16.46 – -8.40	**<0.001**
year_knot13:factor(imd_quintile)2	15.65	-2.28 – 33.58	0.087
year_knot13:factor(imd_quintile)3	3.89	-14.04 – 21.83	0.671
year_knot13:factor(imd_quintile)4	20.50	2.57 – 38.43	**0.025**
year_knot13:factor(imd_quintile)5	24.14	6.20 – 42.07	**0.008**
**Random Effects**			
σ^2	34654.53		
$\tau_{00\ LA\_name}$	3953.12		

For peer review only - http://bmjopen.bmj.com/site/about/guidelines.xhtml

ICC $_{LA\_name}$	0.10
Observations	5832
Marginal R^2 / Conditional R^2	0.159 / 0.245

Web appendix 6: Residual diagnostics for local authority level analysis

The residuals from our longitudinal model plotted below. The residuals are normally distributed.

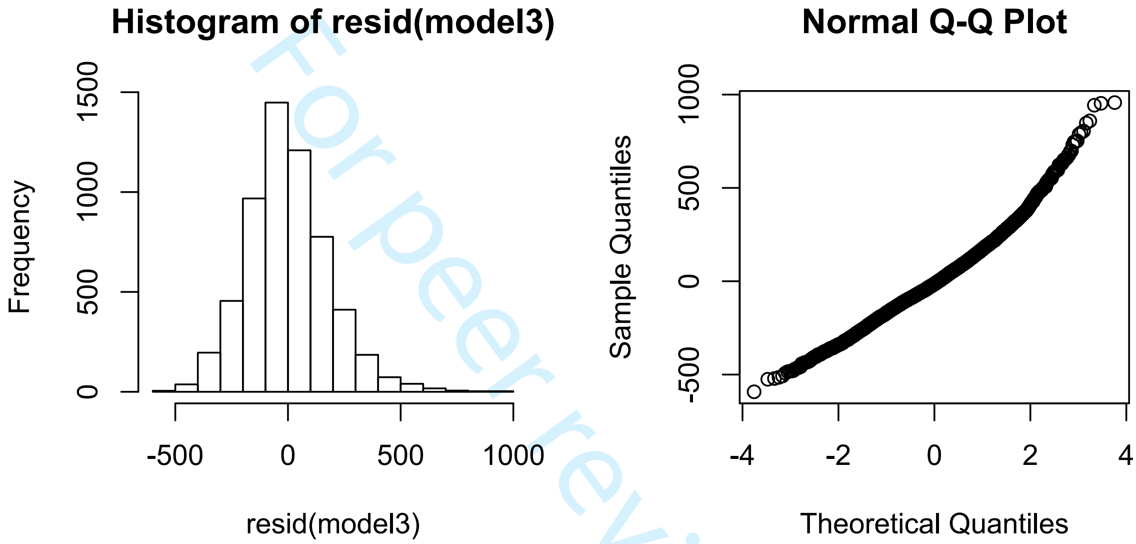

Web appendix 7: Modelling strategy for the fixed effects level analysis

Our cross-regional fixed effects model is as follows.

$Y_{ij} = \beta_1\text{Child Poverty}_{ij} + \boldsymbol{\beta}_2\text{Year} + \mu_i + Z_{ij}$

Let Y_{ij} is the infant mortality rate in the jth year in the ith English region,

Child Poverty$_{ij}$ is the child poverty rate in the jth year in the ith English region.

Year is a set of dummy variables for each year from 2000 to 2017

μ are regional fixed effects, and Z_{ij} are independent copies of a normally distributed random error term.

Web appendix 8: Residual diagnostics for regional fixed effects analysis

For peer review only - http://bmjopen.bmj.com/site/about/guidelines.xhtml

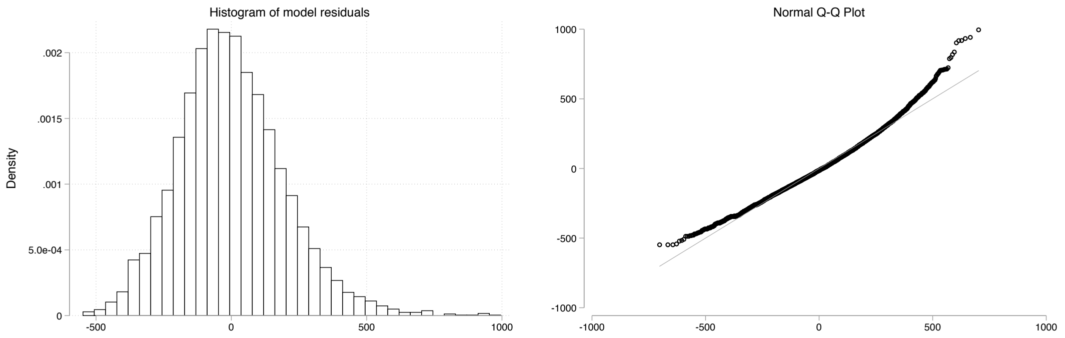

Web appendix 9: Estimates from table 1 plotted with their confidence intervals

Plotting the estimates for annual change (deaths per 100,000) in infant mortality 2014-2017 relative to previous trend (2000-2013) and their confidence intervals by small area deprivation quintile (most deprived=quintile 5) suggests that the data are compatible with a monotonic dose response relationship with deprivation.

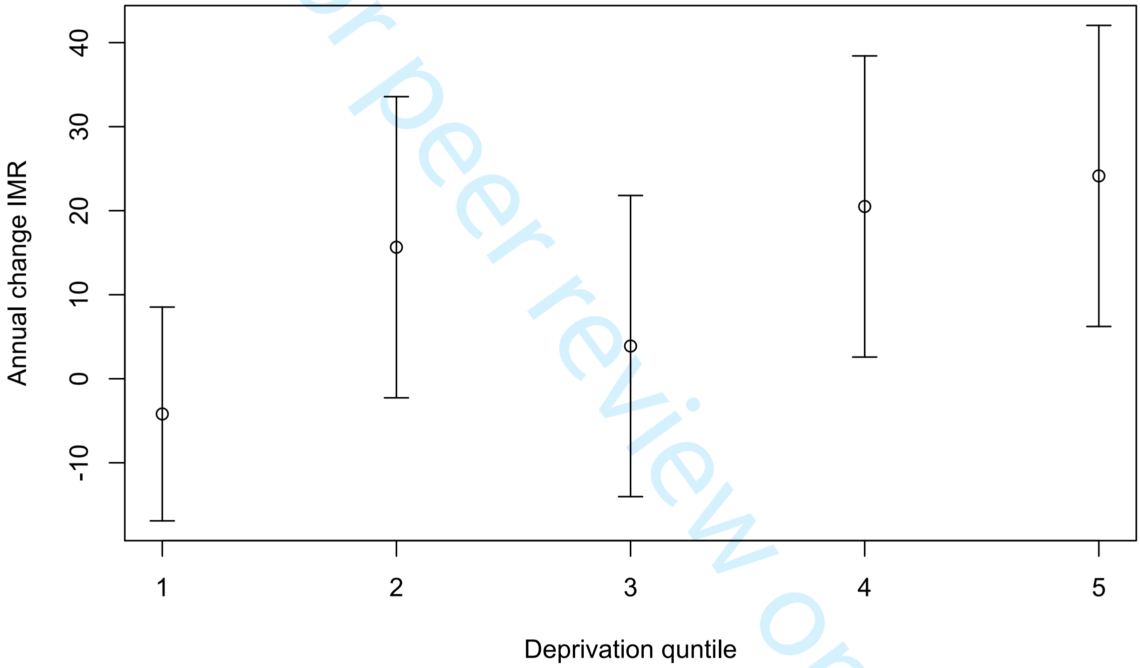

For peer review only

For peer review only - http://bmjopen.bmj.com/site/about/guidelines.xhtml

Web appendix 10: Neonatal mortality and post-neonatal mortality trend by deprivation quintile of local authority district, 2000-2017, with 95% binomial confidence intervals.
A neonatal death is defined as a death during the first 28 days of life (0-27 days); post-neonatal between 28 and 364 days after birth.

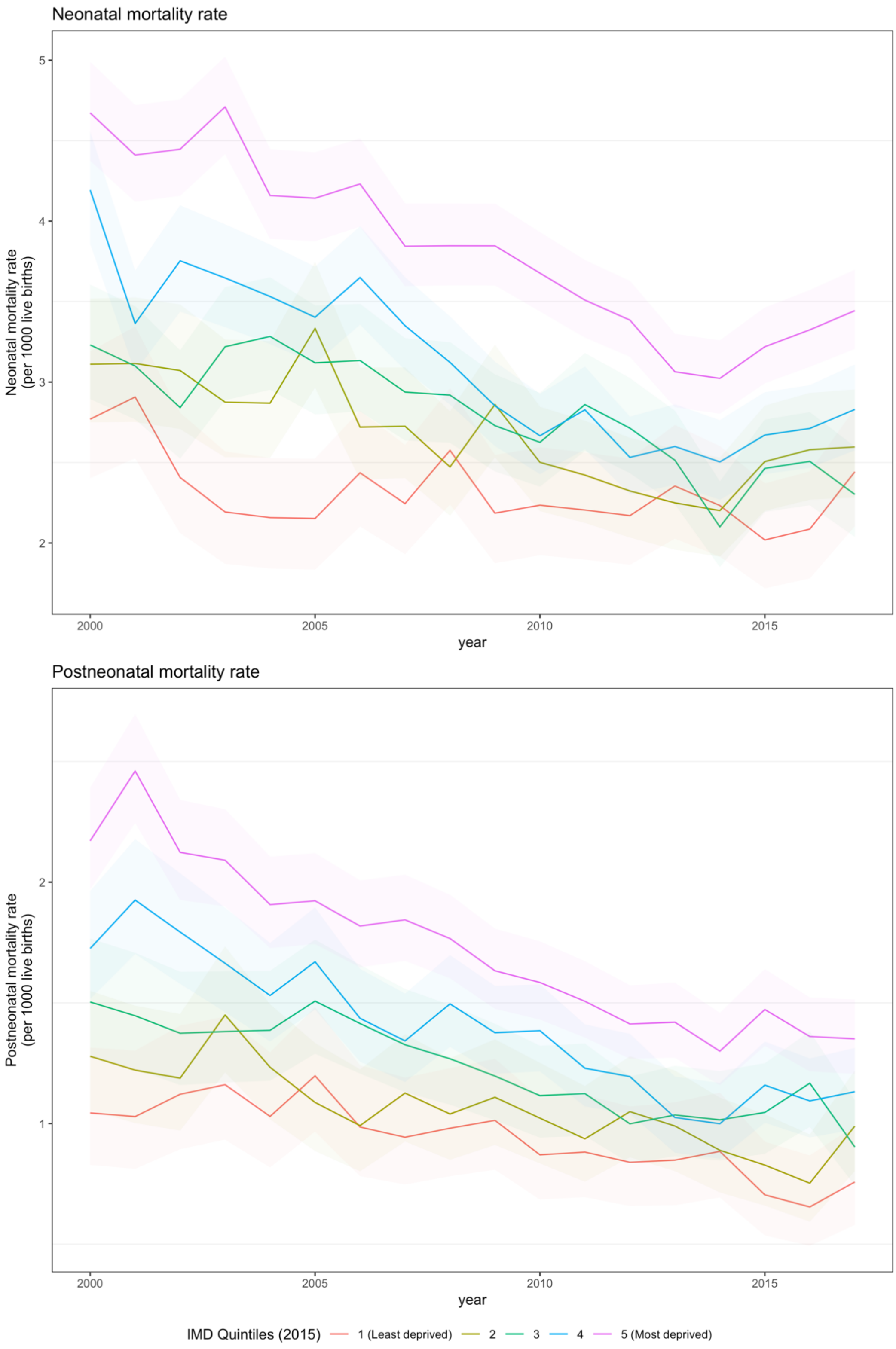

STROBE Statement—checklist of items that should be included in reports of observational studies

	Item No	Recommendation
**Title and abstract**	1	(*a*) Indicate the study's design with a commonly used term in the title or the abstract - Done : Title: **Child poverty and inequalities in infant mortality in England 2000-17: time trend analysis**
		(*b*) Provide in the abstract an informative and balanced summary of what was done and what was found – Done – see ABSTRACT page 2
**Introduction**		
Background/rationale	2	Explain the scientific background and rationale for the investigation being reported. - Done see BACKGROUND page 4
Objectives	3	State specific objectives, including any prespecified hypotheses Done see Abstract and BACKGROUND- "We therefore investigate the extent to which increases in child poverty may have led to increased infant mortality in recent years. Firstly, we investigate whether infant mortality increased more in those parts of the country with the greater numbers of people on a low income or receiving low income related welfare benefits. Secondly, we estimate whether there was a significant change in the trend in infant mortality in those areas and the timing of this change. Thirdly, we exploit regional differences in rates of infant mortality and child poverty to estimate the extent to which those regions with greater increases in child poverty experience greater increases in infant mortality. We use these estimates to calculate the proportion of recent infant mortality increases that are potentially explained by increases in child poverty." Page 5
**Methods**		
Study design	4	Present key elements of study design early in the paper – Done – see METHODS page 5
Setting	5	Describe the setting, locations, and relevant dates, including periods of recruitment, exposure, follow-up, and data collection. Done – see METHODS➔ Setting and data sources page 5
Participants	6	(*a*) *Cohort study*—Give the eligibility criteria, and the sources and methods of selection of participants. Describe methods of follow-up – NA – participants are the entire population<br>*Case-control study*—Give the eligibility criteria, and the sources and methods of case ascertainment and control selection. Give the rationale for the choice of cases and controls- NA<br>*Cross-sectional study*—Give the eligibility criteria, and the sources and methods of selection of participants - NA
		(*b*) *Cohort study*—For matched studies, give matching criteria and number of exposed and unexposed- NA<br>*Case-control study*—For matched studies, give matching criteria and the number of controls per case - NA

For peer review only - http://bmjopen.bmj.com/site/about/guidelines.xhtml

Variables	7	Clearly define all outcomes, exposures, predictors, potential confounders, and effect modifiers. Give diagnostic criteria, if applicable. Done see METHODS➔ Data sources page 5
Data sources/ measurement	8*	For each variable of interest, give sources of data and details of methods of assessment (measurement). Describe comparability of assessment methods if there is more than one group. Done see METHODS➔ Data sources page 5
Bias	9	Describe any efforts to address potential sources of bias- Done see METHODS➔ analysis page 6-7 – fixed effect approach
Study size	10	Explain how the study size was arrived at. NOTE: the study size is the entire population of England, analysed as aggregate data for 324 local authorities and 9 regions.
Quantitative variables	11	Explain how quantitative variables were handled in the analyses. If applicable, describe which groupings were chosen and why. Done see METHODS➔ analysis - page 5 – "Local authorities were grouped in 5 categories (quintiles) based on the income deprivation score of the 2015 Indices of Multiple Deprivation."
Statistical methods	12	(a) Describe all statistical methods, including those used to control for confounding. Done- Done see METHODS➔ analysis- page 6 & 7
		(*b*) Describe any methods used to examine subgroups and interactions Done see METHODS➔ analysis- page 6 &7 – "To do this we used the local authority panel data to estimate a segmented mixed effects regression model for infant mortality including two linear spline terms for time interacted with income deprivation quintile, with a breakpoint indicating the change in trend."
		(*c*) Explain how missing data were addressed- As the measures are based on the whole population this is not relevant. There was no missing data.
		(*d*) *Cohort study*—If applicable, explain how loss to follow-up was addressed - NA<br>*Case-control study*—If applicable, explain how matching of cases and controls was addressed- NA<br>*Cross-sectional study*—If applicable, describe analytical methods taking account of sampling strategy- NA
		(*e*) Describe any sensitivity analyses. Done see robustness tests – page 7. And Web appendix.

Continued on next page

For peer review only - http://bmjopen.bmj.com/site/about/guidelines.xhtml

**Results**		
Participants	13*	(a) Report numbers of individuals at each stage of study—eg numbers potentially eligible, examined for eligibility, confirmed eligible, included in the study, completing follow-up, and analysed- NA
		(b) Give reasons for non-participation at each stage- NA
		(c) Consider use of a flow diagram- NA
Descriptive data	14*	(a) Give characteristics of study participants (eg demographic, clinical, social) and information on exposures and potential confounders –NA
		(b) Indicate number of participants with missing data for each variable of interest- There were no missing data
		(c) *Cohort study*—Summarise follow-up time (eg, average and total amount)- NA
Outcome data	15*	*Cohort study*—Report numbers of outcome events or summary measures over time- NA
		Case-control study—Report numbers in each exposure category, or summary measures of exposure- NA
		Cross-sectional study—Report numbers of outcome events or summary measures- NA
Main results	16	(*a*) Give unadjusted estimates and, if applicable, confounder-adjusted estimates and their precision (eg, 95% confidence interval). Make clear which confounders were adjusted for and why they were included- Done: Unadjusted estimates -see Figure 1 and in web appendix 4, Adjusted estimates in table 1
		(*b*) Report category boundaries when continuous variables were categorized- NA
		(*c*) If relevant, consider translating estimates of relative risk into absolute risk for a meaningful time period- All results are given as both and relative absolute change
Other analyses	17	Report other analyses done—eg analyses of subgroups and interactions, and sensitivity analyses. Done see RESULTS ➔ robustness tests page 8 and web appendix 4.
**Discussion**		
Key results	18	Summarise key results with reference to study objectives. Done see discussion page 8
Limitations	19	Discuss limitations of the study, taking into account sources of potential bias or imprecision. Discuss both direction and magnitude of any potential bias- Done see discussion page 8
Interpretation	20	Give a cautious overall interpretation of results considering objectives, limitations, multiplicity of analyses, results from similar studies, and other relevant evidence- Done see discussion page 8-10
Generalisability	21	Discuss the generalisability (external validity) of the study results. Done see discussion page 10
**Other information**		
Funding	22	Give the source of funding and the role of the funders for the present study and, if applicable, for the original study on which the present article is based- Funding sources given page 11.

*Give information separately for cases and controls in case-control studies and, if applicable, for exposed and unexposed groups in cohort and cross-sectional studies.

Note: An Explanation and Elaboration article discusses each checklist item and gives methodological background and published examples of transparent reporting. The STROBE checklist is best used in conjunction with this article (freely available on the Web sites of PLoS Medicine at http://www.plosmedicine.org/, Annals of Internal Medicine at http://www.annals.org/, and Epidemiology at http://www.epidem.com/). Information on the STROBE Initiative is available at www.strobe-statement.org.

For peer review only - http://bmjopen.bmj.com/site/about/guidelines.xhtml

BMJ Open

Assessing the impact of rising child poverty on the unprecedented rise in infant mortality in England 2000-17: time trend analysis

Journal:	*BMJ Open*
Manuscript ID	bmjopen-2019-029424.R2
Article Type:	Research
Date Submitted by the Author:	12-Jul-2019
Complete List of Authors:	Taylor-Robinson, David; University of Liverpool, Department of Public Health and Policy<br>Lai, Eric; University of Liverpool, Public Health and Policy<br>Wickham, S; University of Liverpool<br>Rose, Tanith; University of Liverpool, Public Health and Policy<br>Norman, Paul; University of Leeds, School of Geography<br>Bambra, Clare; Newcastle University, Institute of Health & Society<br>Whitehead, Margaret; University of Liverpool, Public Health<br>Barr, Ben ; University of Liverpool, Department Public Health and Policy
**Primary Subject Heading**:	Public health
Secondary Subject Heading:	Epidemiology, Paediatrics, Health policy
Keywords:	EPIDEMIOLOGY, PUBLIC HEALTH, Community child health < PAEDIATRICS, Health policy < HEALTH SERVICES ADMINISTRATION & MANAGEMENT

SCHOLARONE™
Manuscripts

Assessing the impact of rising child poverty on the unprecedented rise in infant mortality in England 2000-17: time trend analysis

David Taylor-Robinson[1], Eric TC Lai[1], Sophie Wickham[1], Tanith Rose[1], Paul Norman[2], Clare Bambra[3], Margaret Whitehead[1], Ben Barr[1]

1. Department of Public Health and Policy, The Farr Institute@HeRC, Waterhouse Building 2nd Floor Block F, University of Liverpool, Liverpool, United Kingdom, L69 3GB.
2. School of Geography & Leeds Institute of Data Analytics, University of Leeds, Woodhouse Lane, Leeds, LS2 9JT
3. Institute of Health & Society, Baddiley-Clark, Richardson Road, Newcastle upon Tyne, NE2 4AX

Corresponding author: Professor David Taylor-Robinson; email: dctr@liv.ac.uk

For peer review only

For peer review only - http://bmjopen.bmj.com/site/about/guidelines.xhtml

Abstract

Objective

To determine whether there were inequalities in the sustained rise in infant mortality in England in recent years and the contribution of rising child poverty to these trends.

Design

Analysis of trends in infant mortality in local authorities grouped into 5 categories (quintiles) based on their level of income deprivation. Fixed effects regression models were used to quantify the association between regional changes in child poverty and regional changes in infant mortality.

Setting

324 English local authorities in 9 English Government Office Regions.

Participants

Live born children under 1 year of age.

Main outcome measure

Infant mortality rate (IMR), defined as the number of deaths in children under 1 year of age per 100,000 live births in the same year.

Results

The sustained and unprecedented rise in infant mortality in England from 2014-2017 was not experienced evenly across the population. In the most deprived local authorities, the previously declining trend in infant mortality reversed and mortality rose, leading to an additional 24 infant deaths per 100,000 live births per year (95% CI 6-42), relative to the previous trend. There was no significant change from the pre-existing trend in the most affluent local authorities. As a result, inequalities in infant mortality increased, with the gap between the most and the least deprived local authority areas widening by 52 deaths per 100,000 births (95% CI 36 to 68). Overall from 2014-2017, there were a total of 572 excess infant deaths (95% CI 200 to 944) compared to what would have been expected based on historical trends. We estimated that each 1% increase in child poverty was significantly associated with an extra 5.8 infant deaths per 100,000 live births (95%CI 2.4 to 9.2). The

For peer review only - http://bmjopen.bmj.com/site/about/guidelines.xhtml

findings suggest that about a third of the increases in infant mortality between 2014 and 2017 can be attributed to rising child poverty 172 deaths 95% CI (74 to 266).

Conclusion

This study provides evidence that the unprecedented rise in infant mortality disproportionately affected the poorest areas of the country, leaving the more affluent areas unaffected. Our analysis also linked the recent increase in infant mortality in England with rising child poverty, suggesting that about a third of the increase in infant mortality from 2014-17 may be attributed to rising child poverty.

For peer review only

For peer review only - http://bmjopen.bmj.com/site/about/guidelines.xhtml

Strengths and limitations of this study

- Use of national level data shows that the unprecedented rise in infant mortality in England has disproportionately affected the poorest areas of the country, leaving the more affluent areas unaffected
- Using linked area level data we show that the recent increase in levels of child poverty was associated with about a third of the extra infant deaths in England in the period 2014-17.
- Limitations include the lack of individual level data, and information on potentially mediating pathways, such as changes in health and social care spending on children that may have contributed.

Introduction

Infant mortality rate (IMR) has risen for the last four years in England, yet the role of increasing levels of child poverty in explaining these trends is unclear. Along with others[1], we raised concern about recent rises in infant mortality in England in two letters to the BMJ.[2,3] We noted that this rise had occurred particularly amongst more disadvantaged children from routine and manual socioeconomic groups. Rising infant mortality is unusual in high income countries, and international data show that infant mortality has continued to decline in most rich countries in recent years.[4] Infant mortality is a sensitive indicator of the changing overall health of societies and, as such, acts as an early warning system for future adverse trends. There is therefore an urgent need to understand this extremely concerning trend in England.

Adverse trends in mortality have occurred across all age groups in England in recent years.[5] Several commentators have suggested that these could be due to austerity policies introduced in recent years including cuts to NHS, local authority and public health services and changes to welfare benefits. [6,7] Since 2010, there have been sustained reductions in the welfare benefits available to families with children including: the abolition of child benefit and child tax credit for the third child or more; reductions in the value of tax credits, below inflation up-rating of most working-age benefits; housing benefit reforms including the under occupancy charge (most commonly referred to as 'bedroom tax'), introduction of universal credit; and household caps on total benefit receipt (regardless of how many children are in the household).[8] These welfare changes have disproportionately affected the most

For peer review only - http://bmjopen.bmj.com/site/about/guidelines.xhtml

For peer review only

deprived local authorities and regions [8] and have led to a rise in child poverty.[9] The impacts of these changes on trends in child health have not been considered. For example, a recent review of mortality increases by Public Health England did not consider the potential causes of increases in infant mortality.[5]

There is strong evidence that increased child poverty leads to deteriorating child health and increased infant deaths[10–14]. Whilst relative child poverty declined between 2007 and 2013, we are now seeing increases: child poverty (defined as living in a household with income below 60% of the median household after housing costs) rose by 2 percentage points between 2014 and 2017 and it is projected to increase further through to 2022.[9] By 2017 there were 4.1 million children in England living in relative poverty amounting to 30% of all English children. This compares to less than 10% of children in European countries such as Austria, Denmark, Finland, Iceland, Norway, Slovenia, Sweden, and Switzerland.[15] The recent rises in infant mortality in England have occurred concurrently with these increases in child poverty (figure 1).

Figure 1: Trends in infant mortality and child poverty in England, 2007-17

In this paper, we investigate whether there were inequalities in the sustained rise in infant mortality in England in recent years and the contribution of rising child poverty to these trends. Firstly, we investigate whether infant mortality increased more in those parts of the country with the highest numbers of people receiving low income related welfare benefits, as these are the areas that are most likely to have been adversely affected by recent changes in welfare policy. We estimate whether there was a significant change in the trend in infant mortality in those areas and the timing of this change. Secondly, we exploit regional differences in trends in infant mortality and child poverty to estimate the extent to which those regions with greater increases in child poverty experienced greater increases in infant mortality. We use these estimates to calculate the proportion of recent infant mortality increases that are potentially explained by increases in child poverty.

For peer review only - http://bmjopen.bmj.com/site/about/guidelines.xhtml

Methods

Annual Vital Statistics data for the number of registered infant deaths (< 1 year of age) and live births for 324 lower tier local authority level (324 local areas) between 2000 to 2017 were obtained from the Office of National Statistics. We chose this period due to data availability and since it captures a contemporary period during which both infant mortality and child poverty rates have changed dramatically. Local authorities were grouped in 5 categories (quintiles) based on the income deprivation score of the 2015 Indices of Multiple Deprivation. This score is a non-overlapping count of the numbers of people in each local authority on a low income and in receipt of means tested benefits as a proportion of the population.[16]

Regional relative child poverty was measured as the proportion of children living in households in each region with less than 60 per cent of contemporary national median household income, using the Households Below Average Income data provided by the Department for Work and Pensions.[17] This is the most widely used measure of poverty within the European Union and recognises that the experience of poverty is relative to standards of living that are considered normal within a society and that these average standards change over time. [14] As this measure not available at the local authority level we used data for the nine government office regions of England. [18] See web appendix 1 for a summary of the dataset.

Patient and Public Involvement

Our study was informed by discussions with children and young people's reference groups in Liverpool, who encouraged us to undertake analyses focussed on developing a better understanding of social factors that shape children and young people's (CYP) lives, such as child poverty. The results of our work are feeding into ongoing discussion with CYP about health inequalities, informing the UNICEF Child Friendly City programme in Liverpool.

Statistical analysis

The statistical analysis proceeded in three steps. Firstly, we assessed descriptive trends in infant mortality between 2000 and 2017 for the five groups of local authorities categorised by

For peer review only - http://bmjopen.bmj.com/site/about/guidelines.xhtml

their level of income deprivation (see above). Secondly, we tested whether there was a statistically significant change in the infant mortality trend during this time, the timing of that change in trend and whether any change in trend differed by the level of income deprivation. To do this we used longitudinal local authority area level data to estimate a segmented mixed effects regression model for infant mortality including two linear spline terms for time interacted with income deprivation quintile, with a breakpoint indicating the change in trend. We used an iterative search procedure to identify which breakpoint provided the best fit for the data, [19,20] in other words identifying at which time point a change in trend occurred. This model included random effects (intercepts) at the local authority level to take account of the longitudinal nature of the data (see appendix for details). Secondly, we used this model to estimate whether there had been a significant change in trend in recent years, whether this had led to widening inequalities between local authority areas based on their level of income deprivation and to estimate the level of excess mortality that was attributable to any change in trend. Excess mortality was estimated as the marginal difference between the observed trend and that which would be predicted from the model if pre-existing trends had continued, using the *margins[21]* commands in Stata version 14

In the third step of the analysis, we assessed the association of changes in regional child poverty and regional infant mortality. As there is potential confounding from unobserved factors that vary between regions, or national trends in unobserved factors that affect all region, we used a fixed effects approach to remove between region differences and national trends.[22,23] This conservative approach involves including dummy variables for each region and year to assess the association between deviations from the average rate of change in poverty and deviations from average rate of change in infant mortality in each region (see web appendix 2-3 for further details). This method means that the estimated association between child poverty and infant mortality cannot be confounded by any time invariant differences between regions or any national trend that effects all regions, providing an estimate that is likely to reflect a causal association. We aligned annual regional child poverty rates for financial years to infant mortality registered in the calendar year in which the financial year ended. In other words, 2016-17 child poverty data was aligned with 2017 infant mortality data, meaning that child poverty measures were lagged by 9 months compared to infant mortality measures. We used robust clustered standard errors to ensure that they are robust to serial correlation in the data. This model was used to estimate the number of child deaths attributable to child poverty increases in recent years, by estimating

For peer review only - http://bmjopen.bmj.com/site/about/guidelines.xhtml

the marginal difference between the observed trend and that which would be predicted from the model if child poverty had not increased *[21]*. All models were estimated using Stata version 14 and R version 3.5.1. As a robustness test we repeated our analysis stratified by neonatal and post-neonatal deaths.

Results

Infant mortality had been falling for all groups of local authority areas since 2000, with greater decreases in the most income deprived areas, reducing inequalities. From 2013, this trend changed, and infant mortality increased particularly in the most income deprived local authority areas (figure 2). In web appendix 4 we have provided a link to a visualisation of the geographical distribution of the IMR data for England.

Figure 2: Infant mortality trend by deprivation quintile of local authority district, 2000-2017, with 95% binomial confidence intervals.

The breakpoint analysis also showed a significant change in trend from 2013 (appendix). Table 1 shows the results of the regression model for trends in infant mortality since that time. From 2013-17 there was a dramatic reversal of the pre-existing trend, with an additional annual increase of 24 deaths per 100,000 live births (95%CI 6 to 42) in the most deprived quintile 5 compared to the 2000-2013 trend. A significant change in trend was also observed for quintile 4, whilst there was no significant change from the previously declining trend in the most affluent quintile. The gap in infant mortality between the most income deprived and most affluent quintile increased by 52 deaths per 100,000 births (95% CI 36 to 68). Based upon these trends we estimated that there were an additional 570 excess deaths (95% CI 200 to 944) in the period 2014-17 than would have been expected if the historical trends had continued.

Table 1: Segmented regression model results for trends in infant mortality at lower tier local authority level 2014-2017 (per 100,000 live births per year)

Level of income deprivation	Annual change (deaths per 100,000) in infant mortality 2014-2017 relative to previous trend (2000-2013) (95% CI)	P value for change in trend from previous period

For peer review only - http://bmjopen.bmj.com/site/about/guidelines.xhtml

Quintile 1 (most affluent)	-4.19	[-16.91,8.53]	0.518
Quintile 2	15.65	[-2.27,33.57]	0.087
Quintile 3	3.89	[-14.03,21.81]	0.67
Quintile 4	20.5	[2.58,38.42]	0.025
Quintile 5 (most deprived)	24.14	[6.22,42.05]	0.008

Estimates based on random effects regression model using LA panel dataset of infant mortality from 2000 to 2017, N= 5,832 local authority years. See web appendix 5-9 for full model output and checks.

In the fixed effects analysis each percentage point change in child poverty was associated with a change in infant mortality of 5.8 deaths per 100,000 live births (95% confidence interval 2.4 to 8.9). From this model we estimate that 172 deaths 95% CI (74 to 266) between 2014 and 2017 were attributable to increases in child poverty, almost a third of the overall rise in infant mortality over that period. Repeating our analysis for neonatal and post-neonatal deaths showed similar results, with a change in trend impacting the most disadvantaged quintiles (see web appendix 10 and 11).

Discussion

Since 2013, infant mortality has increased in England and there have been an additional 570 infant deaths over 4 years (2014 to 2017) compared to what would have been expected based on recent historical trends. These excess deaths have largely occurred in the most disadvantaged areas, increasing inequalities. Our study estimated that the recent increase in levels of child poverty was associated with 172 (about a third) of the extra infant deaths in England in the period 2014-17.

Limitations of the study

Before evaluating the implications of our findings, we note several important limitations. Firstly, due to absence of individual level data, we undertook an observational analysis at the population level. Lack of data on levels of child poverty at a small area level meant that we undertook our analysis of the effects of child poverty using child poverty estimates for 9 regions within England. This meant we could take advantage of regional differences in trends over time, and use a fixed effects approach to adjust for time invariant confounding variables. We were not able, however, to estimate the effect of increases in child poverty on trends in

For peer review only - http://bmjopen.bmj.com/site/about/guidelines.xhtml

For peer review only

infant mortality for groups of local authorities within these regions. As our main analysis was based on aggregate data, however, we cannot identify whether the additional infant deaths were in the same families who have experienced a rise in child poverty. It is also possible that the association between the child poverty and infant mortality in our analysis was due to trends in unobserved time-varying confounding factors that varied between regions.

Secondly, we measured child poverty using the relative poverty measure used by the Department for Work and Pensions in England, because it is used internationally, as well as in the UK. Furthermore, whether or not relative measures of income poverty effectively reflect children's life chances has been the focus of policy debates in the UK.[24] This measure, however, is a simplification of underlying income trajectories whereby families with children move in and out of poverty over time. Furthermore, the measure we have used is likely to underestimate the extent of the experience of poverty in England. A recent report published by the Social Metrics Commission outlining a new measure of poverty which extends beyond income-based poverty, suggests that there are 4.5 million children living (32.6%) in poverty in the UK.[25] In addition, the Joseph Rowntree Foundation's (JRF) has shown that the proportion of children living below the Minimum Income Standard has increased from 39% to 45% (an increase of about 1 million) between 2008-09 and 2014-15.[26] Further studies could usefully explore the relationship between a continuous measure of income and aspects of child health using individual level data, in order to identify any policy relevant threshold effects.

Thirdly, we did not have data on cause of death, and were not able to investigate the factors mediating the association between rising child poverty and rising infant mortality, such as birth characteristics (for example preterm birth, maternal age and maternal smoking) and post-natal factors (for example breastfeeding and child care in the first year of life).[27,28] Fourthly, it is important that further research investigates the potential role of parental characteristics, gestational age and other risk factors for child mortality in explaining the observed trends, in addition to assessing how changes in health and social care spending on children may have contributed. [6,7]

Comparison with previous studies

To our knowledge this is the first study to explore both the inequalities in the unprecedented rise in infant mortality in England on the basis of area deprivation and the

For peer review only - http://bmjopen.bmj.com/site/about/guidelines.xhtml

reasons behind this rise. Whilst the UK government recently commissioned a report into adverse mortality trends, infant mortality was not considered in any detail. [5] Our analysis corroborates our previous analysis of trends in infant mortality on the basis of parental occupational social class, which suggested that IMR was rising particularly in the most disadvantaged social groups.[3,29] A recent report by the Royal College of Paediatrics and Child Health (RCPCH) also noted the reversal of over 100 year of declining infant mortality in England and Wales, and that rises in infant mortality were most striking in the deprived portion of the population. [27] The RCPCH further assesses various scenarios of infant mortality, for instance showing that even if infant mortality begins to decline again at its previous rate, rates will be 80% higher than the EU15+ average in 2030. Our study uses the most up-to date infant mortality data, and suggests that rather than just stalling, infant mortality is rising sharply in the most disadvantaged areas.

In addition to increasing inequalities in infant mortality, social inequalities in numerous aspects of health in England appear to be rising.[7,19,30,31] Barr and colleagues demonstrated a reversal in trends in inequalities in life expectancy.[19] Two analyses from the Global Burden of Diseases collaboration have shown both a stagnation in the improvement in life expectancy in England; and increasing inequalities since 2010.[30,31] Furthermore Bennett et al identify deaths in children younger than 5 years as a major contributor to inequality in life expectancy.[30] A further analysis has demonstrated an increase in the North-South divide in health in England.[7]

Our study shows that rising income poverty may be contributing to rising infant mortality. This finding is likely to be generalisable to other high income settings, since numerous studies conducted in Western Europe and the USA have shown an association between social disadvantage and infant mortality. [32–35] Furthermore, numerous studies have suggested that there is a causal link between increasing child poverty and a deterioration in various aspects of child health and development [10–14] For example, a recent systematic review by Cooper and Stewart concluded that "poorer children have worse cognitive, social-behavioural and health outcomes in part because they are poorer, and not just because poverty is correlated with other household and parental characteristics." Their review demonstrated clear causal links between income poverty and a range of child health outcomes, including only studies that applied quasi-experimental designs.[13,36] Komro and colleagues, for example, used a difference-in-

For peer review only - http://bmjopen.bmj.com/site/about/guidelines.xhtml

differences research design to assess the impact of state-level minimum wage in the USA on infant mortality. The authors show that each dollar increase in the minimum wage above the federal level was associated with a 1% to 2% decrease in low birth weight births and a 4% decrease in postneonatal mortality. [37] Additionally, the results of a longitudinal analysis of social welfare expenditure data from 19 Organisation for Economic Co-operation and Development (OECD) countries over the time period from 1980 to 2010, showed that cash benefits for families have positive effects on reducing infant mortality, with a $10 increase in family cash allowances per child predicting a reduction in infant mortality by 4% (p = .007).[12] Previous research has shown that expansions to social security nets and public services have decreased inequalities in life expectancy [19], infant mortality rates [38], and mortality amenable to healthcare.[39] Robinson et al, for example, found that the English health inequalities strategy (2000-2010) was associated with decreases in inequalities in infant mortality in England. [38] Similarly, Krieger et al found that the 1960s ‘War on Poverty’ led to decreases in inequalities in IMR in the USA.[40]

Policy implications

Our analysis has important implications in the context of the projected ongoing increase in levels of child poverty in the UK. For the first time in nearly 40 years there has been a sustained increase in infant mortality in the poorest areas. English regions with the largest rises in child poverty have had the largest increases in infant mortality. Our analysis suggests that the weakened social protection safety net - leading to rising levels of child poverty – may be contributing to this rise in mortality in the most disadvantaged infants. Our analysis suggests that the rise in child poverty explains approximately a third of this rise. This could be an underestimate of the impact if the measures of child poverty used are an underestimate of the true increase in disadvantage facing children (see above). It may also be the case that other policy changes occurring at this time such as the real terms cuts to NHS, local authority children’s services, social care and public health budgets –are also be contributing to the rise in mortality as has been suggested elsewhere. [6,7]

Infant mortality is an uncommon event and represents the most severe tip of the iceberg in terms of the impact of social conditions on health. It can, however, act as an early warning system or litmus test of the overall health of societies. Rising poverty is likely to be having a

For peer review only - http://bmjopen.bmj.com/site/about/guidelines.xhtml

myriad of adverse impacts on other aspects of child health that will have repercussions across the lifecourse.[36] In the context of increasing health inequalities in England, policies that reduce poverty and social inequalities, investing in child health and social care are likely to reduce the occurrence of infant mortality and that of many other adverse child health outcomes.[30]

This rise in mortality in the most disadvantaged children is unprecedented and requires urgent action by national and local governments and the health and social care system. It is likely that the rise in child poverty is an important factor contributing to this trend. As the United Nations (UN) has recently highlighted, rising poverty in the UK is a political choice[41] and it is time for the government to reverse this trend establishing a welfare system that protects children from poverty.

What is already known on this topic

In 2013, infant mortality rates began to rise in England, reversing a long-term decline. It is not clear whether this increase affected all sections of the population evenly and how much can be attributed to rising levels of child poverty.

What this study adds

The sustained increase in infant mortality in England from 2013-17 has occurred in the most disadvantaged areas, while more affluent areas continued in their previous declining trend. This rise in infant mortality in disadvantaged areas is partially explained by rising child poverty.

For peer review only

Funding: DTR and EL are funded by the MRC on a Clinician Scientist Fellowship (MR/P008577/1). SW is supported by a Wellcome Trust Society and Ethics fellowship (grant number 200335/Z/15/Z). BB and TR are supported by the National Institute for Health Research (NIHR) Collaboration for Leadership in Health Research and Care (CLAHRC NWC) and the NIHR School for Public Health Research.

The MRC, Wellcome Trust and NIHR had no role in the study design, data collection and analysis, decision to publish, or preparation of the manuscript. This report is independent research arising from research supported by the MRC, Wellcome Trust and NIHR. The views expressed in this publication are those of the authors and not necessarily those of the NHS, the NIHR, or the Department of Health and Social Care.

For peer review only

Competing interest statement

All authors have completed the ICMJE uniform disclosure format www.icmje.org/coi_disclosure.pdf and declare: DTR and EL are funded by the MRC on a Clinician Scientist Fellowship (MR/P008577/1). SW is supported by a Wellcome Trust Society and Ethics fellowship (grant number 200335/Z/15/Z). BB and TR are supported by the National Institute for Health Research (NIHR) Collaboration for Leadership in Health Research and Care (CLAHRC NWC); no financial relationships with any organisations that might have an interest in the submitted work in the previous three years; no other relationships or activities that could appear to have influenced the submitted work.

Contributorship statement

DTR is lead author and guarantor. DTR and BB planned the study and led the drafting and revising of the manuscript. DTR, BB and EL conducted the analysis. DTR, BB, EL, SW, TR, PN, CB and MMW contributed to interpretation of the data, drafting of the manuscript, and revisions. All authors agreed the submitted version of the manuscript.

Transparency declaration

DTR is the guarantor and affirms that the manuscript is an honest, accurate, and transparent account of the study being reported; that no important aspects of the study have been omitted;

For peer review only - http://bmjopen.bmj.com/site/about/guidelines.xhtml

and that any discrepancies from the study as planned (and, if relevant, registered) have been explained.

Ethics committee approval

None required

Data sharing

Statistical code available on request from lead author

Licence statement

The Corresponding Author has the right to grant on behalf of all authors and does grant on behalf of all authors, a worldwide licence (http://www.bmj.com/sites/default/files/BMJ%20Author%20Licence%20March%202013.doc) to the Publishers and its licensees in perpetuity, in all forms, formats and media (whether known now or created in the future), to i) publish, reproduce, distribute, display and store the Contribution, ii) translate the Contribution into other languages, create adaptations, reprints, include within collections and create summaries, extracts and/or, abstracts of the Contribution and convert or allow conversion into any format including without limitation audio, iii) create any other derivative work(s) based in whole or part on the on the Contribution, iv) to exploit all subsidiary rights to exploit all subsidiary rights that currently exist or as may exist in the future in the Contribution, v) the inclusion of electronic links from the Contribution to third party material where-ever it may be located; and, vi) licence any third party to do any or all of the above. All research articles will be made available on an open access basis (with authors being asked to pay an open access fee—see http://www.bmj.com/about-bmj/resources-authors/forms-policies-and-checklists/copyright-open-access-and-permission-reuse). The terms of such open access shall be governed by a Creative Commons licence—details as to which Creative Commons licence will apply to the research article are set out in our worldwide licence referred to above.

For peer review only - http://bmjopen.bmj.com/site/about/guidelines.xhtml

References

1 Hiam L, Dorling D. Government's misplaced prevention agenda. *BMJ* 2018;**363**:k5134. doi:10.1136/bmj.k5134

2 Taylor-Robinson D, Barr B. Death rate now rising in UK's poorest infants. *BMJ* 2017;**357**:j2258. doi:10.1136/bmj.j2258

3 Death rate continues to rise for poorest infants in England and Wales. *The BMJ* Published Online First: 13 September 2018.https://www.bmj.com/content/360/bmj.k1090/rr-2 (accessed 17 Sep 2018).

4 Health status - Infant mortality rates - OECD Data. theOECD. http://data.oecd.org/healthstat/infant-mortality-rates.htm (accessed 23 Nov 2018).

5 Public Health England. A review of recent trends in mortality in England. London: : PHE 2018.

6 Bennett JE, Li G, Foreman K, *et al.* The future of life expectancy and life expectancy inequalities in England and Wales: Bayesian spatiotemporal forecasting. *The Lancet* 2015;**386**:163–70. doi:10.1016/S0140-6736(15)60296-3

7 Kontopantelis E, Buchan I, Webb RT, *et al.* Disparities in mortality among 25–44-year-olds in England: a longitudinal, population-based study. *The Lancet Public Health* 2018;**0**. doi:10.1016/S2468-2667(18)30177-4

8 Beatty C, Fothergill S. The uneven impact of welfare reform : the financial losses to places and people. 2016. doi:10.7190/cresr.2017.5563239352

9 Hood A, Waters T, Institute for Fiscal Studies (Great Britain), *et al. Living standards, poverty and inequality in the UK: 2016-17 to 2021-22*. 2017.

10 Wang H, Liddell CA, Coates MM, *et al.* Global, regional, and national levels of neonatal, infant, and under-5 mortality during 1990–2013: a systematic analysis for the Global Burden of Disease Study 2013. *The Lancet* 2014;**384**:957–79. doi:10.1016/S0140-6736(14)60497-9

11 Zylbersztejn A, Gilbert R, Hjern A, *et al.* Child mortality in England compared with Sweden: a birth cohort study. *The Lancet* 2018;**0**. doi:10.1016/S0140-6736(18)30670-6

12 Shim J. Social Welfare Expenditures and Infant Mortality. *Soc Work Public Health* 2015;**30**:567–77. doi:10.1080/19371918.2015.1073648

13 Cooper K, Stewart K. *Does Money Affect Children's Outcomes?: A Systematic Review*. Joseph Rowntree Foundation 2013.

14 Wickham S, Anwar E, Barr B, *et al.* Poverty and child health in the UK: using evidence for action. *Archives of disease in childhood* Published Online First: 2016. doi:10.1136/archdischild-2014-306746

15 OECD. Child_Poverty. https://www.oecd.org/els/CO_2_2_Child_Poverty.pdf (accessed 17 Dec 2018).

For peer review only - http://bmjopen.bmj.com/site/about/guidelines.xhtml

16 English indices of deprivation 2015. GOV.UK. https://www.gov.uk/government/statistics/english-indices-of-deprivation-2015 (accessed 10 Dec 2018).

17 Households below average income (HBAI) statistics. GOV.UK. https://www.gov.uk/government/collections/households-below-average-income-hbai--2 (accessed 23 Nov 2018).

18 ONS. Administrative geography. https://www.ons.gov.uk/methodology/geography/ukgeographies/administrativegeography/england (accessed 14 Dec 2018).

19 Barr B, Higgerson J, Whitehead M. Investigating the impact of the English health inequalities strategy: time trend analysis. *BMJ* 2017;**358**:j3310. doi:10.1136/bmj.j3310

20 Crawley M. *An Introduction to Statistics using R*. Chichechester: : John Wiley and Sons 2005.

21 Marginal analysis | Stata. https://www.stata.com/features/overview/marginal-analysis/ (accessed 18 Dec 2018).

22 Barr B, Taylor-Robinson D, Scott-Samuel A, *et al.* Suicides associated with the 2008-10 economic recession in England: time trend analysis. *BMJ* 2012;**345**:e5142. doi:10.1136/bmj.e5142

23 Barr B, Taylor-Robinson D, Stuckler D, *et al.* 'First, do no harm': are disability assessments associated with adverse trends in mental health? A longitudinal ecological study. *J Epidemiol Community Health* 2015;:jech-2015-206209. doi:10.1136/jech-2015-206209

24 FPH. APPG Inquiry: Child Poverty and Health. https://www.fph.org.uk/media/1374/appg-health-in-all-policies-inquiry-into-child-poverty-and-health2.pdf (accessed 14 Dec 2018).

25 Stroud P. The final report of the Social Metrics Commission: A new measure of poverty for the UK. https://lif.blob.core.windows.net/lif/docs/default-source/default-library/legj6470-measuring-poverty-full_report-181004-web.pdf?sfvrsn=0

26 Households below a Minimum Income Standard: 2008/09 to 2014/15. JRF. 2017.https://www.jrf.org.uk/report/households-below-minimum-income-standard-200809-201415 (accessed 10 Dec 2018).

27 Child health in England in 2030: comparisons with other wealthy countries | RCPCH. https://www.rcpch.ac.uk/resources/child-health-england-2030-comparisons-other-wealthy-countries (accessed 10 Dec 2018).

28 Sidebotham P, Fraser J, Covington T, *et al.* Understanding why children die in high-income countries. *Lancet* 2014;**384**:915–27. doi:10.1016/S0140-6736(14)60581-X

29 Taylor-Robinson D, Barr B. Death rate now rising in UK's poorest infants. *BMJ* 2017;**357**:j2258. doi:10.1136/bmj.j2258

For peer review only - http://bmjopen.bmj.com/site/about/guidelines.xhtml

30 Bennett JE, Pearson-Stuttard J, Kontis V, *et al.* Contributions of diseases and injuries to widening life expectancy inequalities in England from 2001 to 2016: a population-based analysis of vital registration data. *The Lancet Public Health* 2018;**3**:e586–97. doi:10.1016/S2468-2667(18)30214-7

31 Steel N, Ford JA, Newton JN, *et al.* Changes in health in the countries of the UK and 150 English Local Authority areas 1990–2016: a systematic analysis for the Global Burden of Disease Study 2016. *The Lancet* 2018;**392**:1647–61. doi:10.1016/S0140-6736(18)32207-4

32 Kim D, Saada A. The social determinants of infant mortality and birth outcomes in Western developed nations: a cross-country systematic review. *Int J Environ Res Public Health* 2013;**10**:2296–335. doi:10.3390/ijerph10062296

33 Weightman AL, Morgan HE, Shepherd MA, *et al.* Social inequality and infant health in the UK: systematic review and meta-analyses. *BMJ Open* 2012;**2**:e000964. doi:10.1136/bmjopen-2012-000964

34 Gisselmann MD. The influence of maternal childhood and adulthood social class on the health of the infant. *Soc Sci Med* 2006;**63**:1023–33. doi:10.1016/j.socscimed.2006.03.015

35 Kihal-Talantikite W, Padilla CM, Lalloué B, *et al.* Green space, social inequalities and neonatal mortality in France. *BMC Pregnancy Childbirth* 2013;**13**:191. doi:10.1186/1471-2393-13-191

36 Cooper K, Stewart K. Money Matters: Does Money Affect Children's Outcomes? A Systematic Review Update. http://sticerd.lse.ac.uk/case/_new/research/money_matters/default.asp

37 Komro KA, Livingston MD, Markowitz S, *et al.* The Effect of an Increased Minimum Wage on Infant Mortality and Birth Weight. *Am J Public Health* 2016;**106**:1514–6. doi:10.2105/AJPH.2016.303268

38 Robinson TJ, Barr B, Brown HW, *et al.* OP18 Evaluating the impact of the english health inequalities strategy on socioeconomic inequalities in the regional infant mortality rate. *J Epidemiol Community Health* 2018;**72**:A9–A9. doi:10.1136/jech-2018-SSMabstracts.18

39 Barr B, Bambra C, Whitehead M. The impact of NHS resource allocation policy on health inequalities in England 2001-11: longitudinal ecological study. *BMJ* 2014;**348**:g3231. doi:10.1136/bmj.g3231

40 Krieger N, Rehkopf DH, Chen JT, *et al.* The fall and rise of US inequities in premature mortality: 1960-2002. *PLoS Med* 2008;**5**:e46. doi:10.1371/journal.pmed.0050046

41 Poverty is a political choice. *The Lancet Public Health* 2018;**3**:e555. doi:10.1016/S2468-2667(18)30243-3

For peer review only - http://bmjopen.bmj.com/site/about/guidelines.xhtml

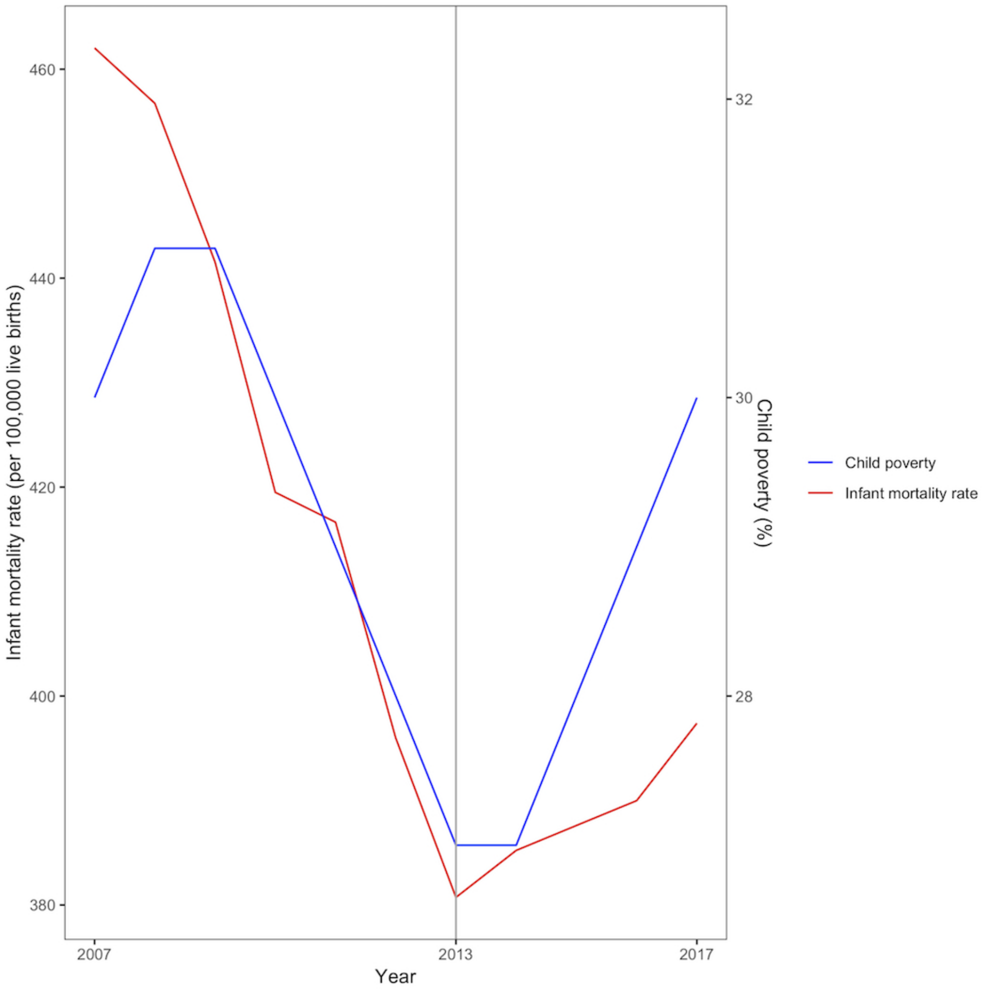

90x90mm (300 x 300 DPI)

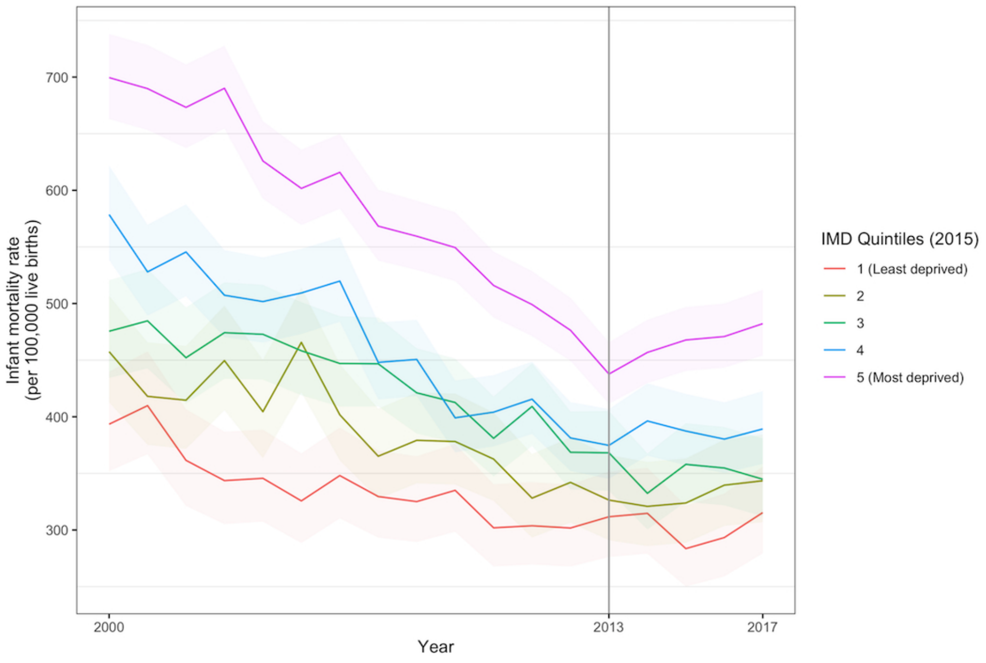

90x60mm (300 x 300 DPI)

Supplementary Material: Child poverty and inequalities in infant mortality in England 2000-17: time trend analysis

Web appendix 1: Summary of dataset for local authority level analysis

Statistic	N	Mean	St. Dev.	Min	Max
Year	5,832	2,008	5	2,000	2,017
birth	5,832	1,975	1,605	222	17,766
death	5,832	9	11	0	169
imd_quintile	5,832	3	1	1	5
year_centred	5,832	8	5	0	17
year_knot13	5,832	1	1	0	4
imr	5,832	418	214	0	1,570

Web appendix 2: Jointpoint regression to identify breakpoint

We therefore investigated empirically whether there was a significant change in the trend in infant mortality between 2000 and 2017. We use an iterative search procedure to identify which breakpoint produced the best fitting mode by comparing all models with these alternative breakpoints at each year from 2001 to 2016, as well as models no breakpoints. We then plotted the Residual Mean Squared Error (RMSE) values from each of these models to identify the model that provided the best fit with the data. In other words we fitted 16 separate models each with a different break points. The figure below shows the RMSE from each of these models – indicating that a breakpoint at 2013 provides the best fitting model compared to all the other alternative break points.

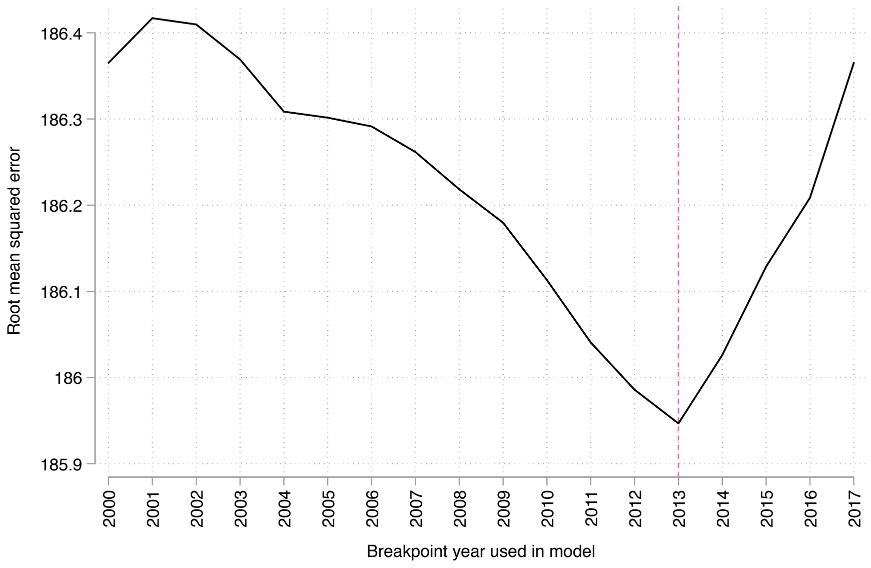

For peer review only

Web appendix 3: Segmented mixed effects regression model for infant mortality including two linear spline terms

We use a segmented mixed effects regression model for the mean response:

$$Y_{ij} = \beta_1 * \text{spline1} + \beta_2 * \text{spline2} + \beta_2 * \text{spline1} * \text{IMDQ} + \beta_4 * \text{spline2} * \text{IMDQ} + U_i + Z_{ij}$$

Let Y_{ij} is the jth repeated measurement (here, IMR) on the ith local area. Spline1 takes the value of year. Spline2 takes the value of *0* for year less than *2013*, taken to be the breakpoint for change in trend*, and year − 2013* for year greater than *2013. This* defines a segmented regression with a change in slope of IMR at year=2013. IMDQ is IMD quintile, coded as a categorical variable. U_i is a random intercept term describing how the average IMR of the ith local area varies about the population-averaged response for all local areas with the same values of the explanatory variables. The model assumes that the U_i are independent copies of a Normally distributed random variable with mean zero and variance v^2. Z_{ij} is an error term that assumes that the Z_{ij} are independent copies of a Normally distributed random variable with mean zero and variance τ^2.

Web appendix 4: Link showing show the distribution of infant mortality across England

https://dblalex.carto.com/builder/c81de0d5-f893-4109-9471-b4b55afa351e/embed

Web appendix 5: Linear mixed effects model results for trends in infant mortality at lower tier local authority level 2000-2017 – full R output with random effects.

	imr		
Predictors	*Estimates*	*CI*	*p*
(Intercept)	377.45	349.94 – 404.96	**<0.001**
year centred	-5.84	-8.70 – -2.98	**<0.001**
year knot 13	-4.19	-16.92 – 8.54	0.519
factor(imd quintile)2	66.13	27.38 – 104.89	**0.001**
factor(imd quintile)3	109.87	71.12 – 148.62	**<0.001**
factor(imd quintile)4	170.67	131.91 – 209.42	**<0.001**
factor(imd quintile)5	292.08	253.33 – 330.83	**<0.001**
year_centred:factor(imd_quintile)2	-2.67	-6.70 – 1.36	0.194
year_centred:factor(imd_quintile)3	-2.56	-6.59 – 1.47	0.213
year_centred:factor(imd_quintile)4	-7.27	-11.30 – -3.24	**<0.001**
year_centred:factor(imd_quintile)5	-12.43	-16.46 – -8.40	**<0.001**
year_knot13:factor(imd_quintile)2	15.65	-2.28 – 33.58	0.087
year_knot13:factor(imd_quintile)3	3.89	-14.04 – 21.83	0.671
year_knot13:factor(imd_quintile)4	20.50	2.57 – 38.43	**0.025**
year_knot13:factor(imd_quintile)5	24.14	6.20 – 42.07	**0.008**
**Random Effects**			
σ^2	34654.53		
$\tau_{00\ \text{LA\_name}}$	3953.12		

ICC $_{LA\_name}$	0.10
Observations	5832
Marginal R^2 / Conditional R^2	0.159 / 0.245

Web appendix 6: Residual diagnostics for local authority level analysis

The residuals from our longitudinal model plotted below. The residuals are normally distributed.

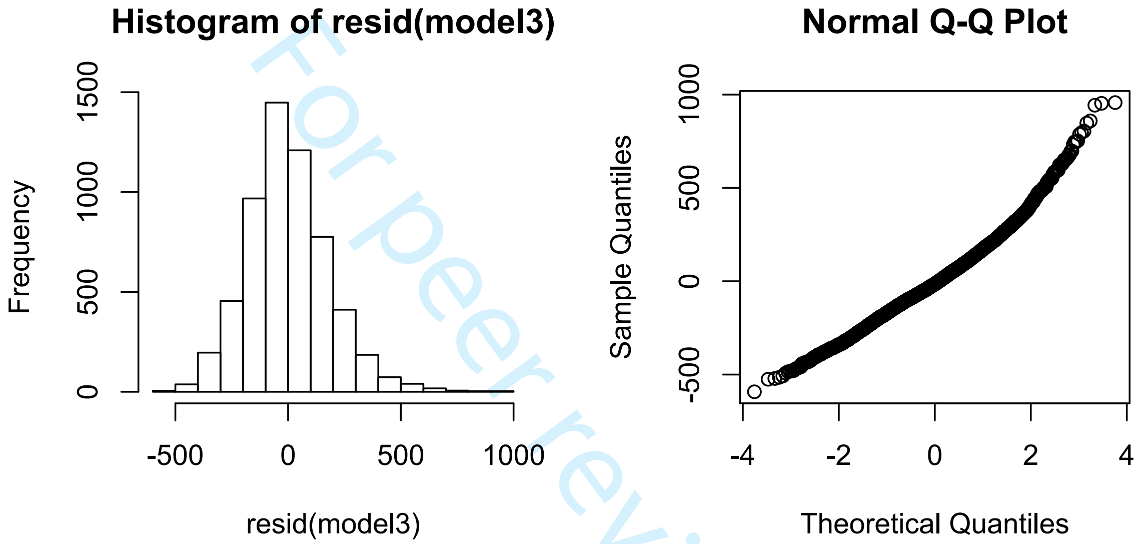

Web appendix 7: Modelling strategy for the fixed effects level analysis

Our cross-regional fixed effects model is as follows.

$Y_{ij} = \beta_1\text{Child Poverty}_{ij} + \boldsymbol{\beta}_2\text{Year} + \mu_i + Z_{ij}$

Let Y_{ij} is the infant mortality rate in the jth year in the ith English region,

Child Poverty$_{ij}$ is the child poverty rate in the jth year in the ith English region.

Year is a set of dummy variables for each year from 2000 to 2017

μ are regional fixed effects, and Z_{ij} are independent copies of a normally distributed random error term.

Web appendix 8: Residual diagnostics for regional fixed effects analysis

For peer review only

For peer review only - http://bmjopen.bmj.com/site/about/guidelines.xhtml

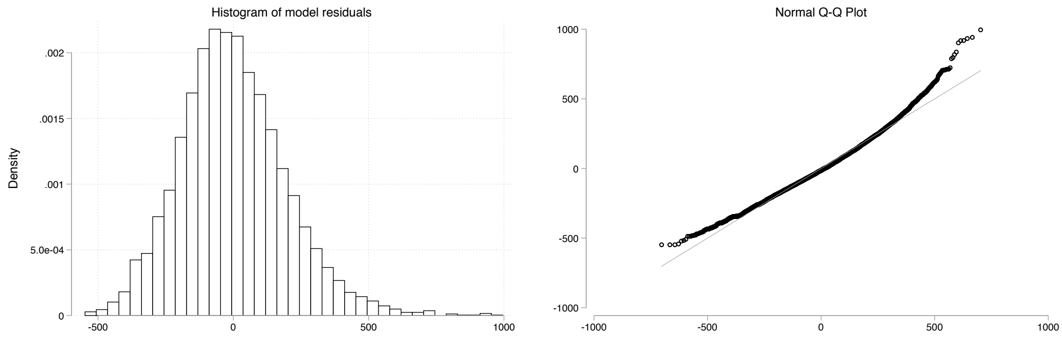

Web appendix 9: Estimates from table 1 plotted with their confidence intervals

Plotting the estimates for annual change (deaths per 100,000) in infant mortality 2014-2017 relative to previous trend (2000-2013) and their confidence intervals by small area deprivation quintile (most deprived=quintile 5) suggests that the data are compatible with a monotonic dose response relationship with deprivation.

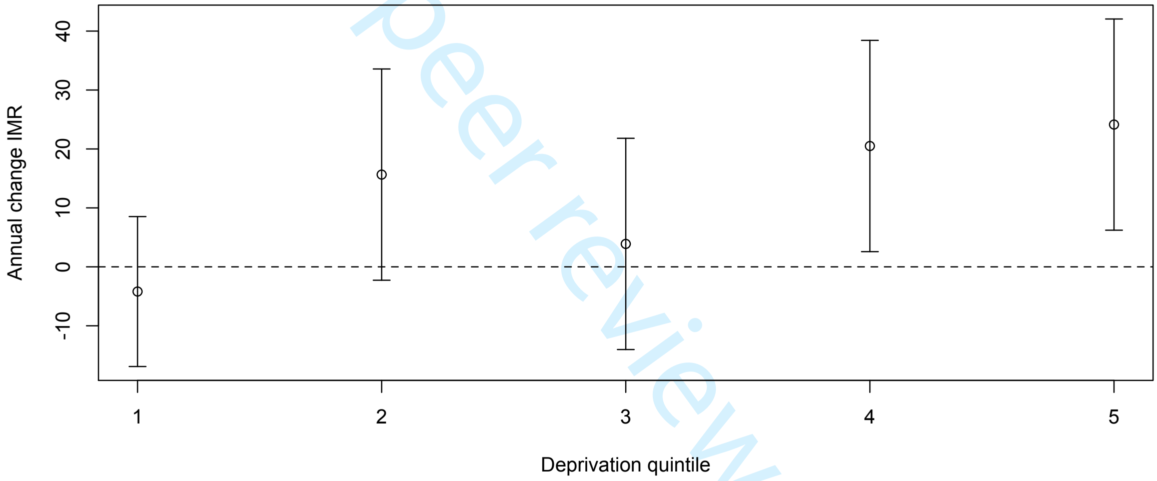

For peer review only

Web appendix 10: Neonatal mortality and post-neonatal mortality trend by deprivation quintile of local authority district, 2000-2017, with 95% binomial confidence intervals.
A neonatal death is defined as a death during the first 28 days of life (0-27 days); post-neonatal between 28 and 364 days after birth.

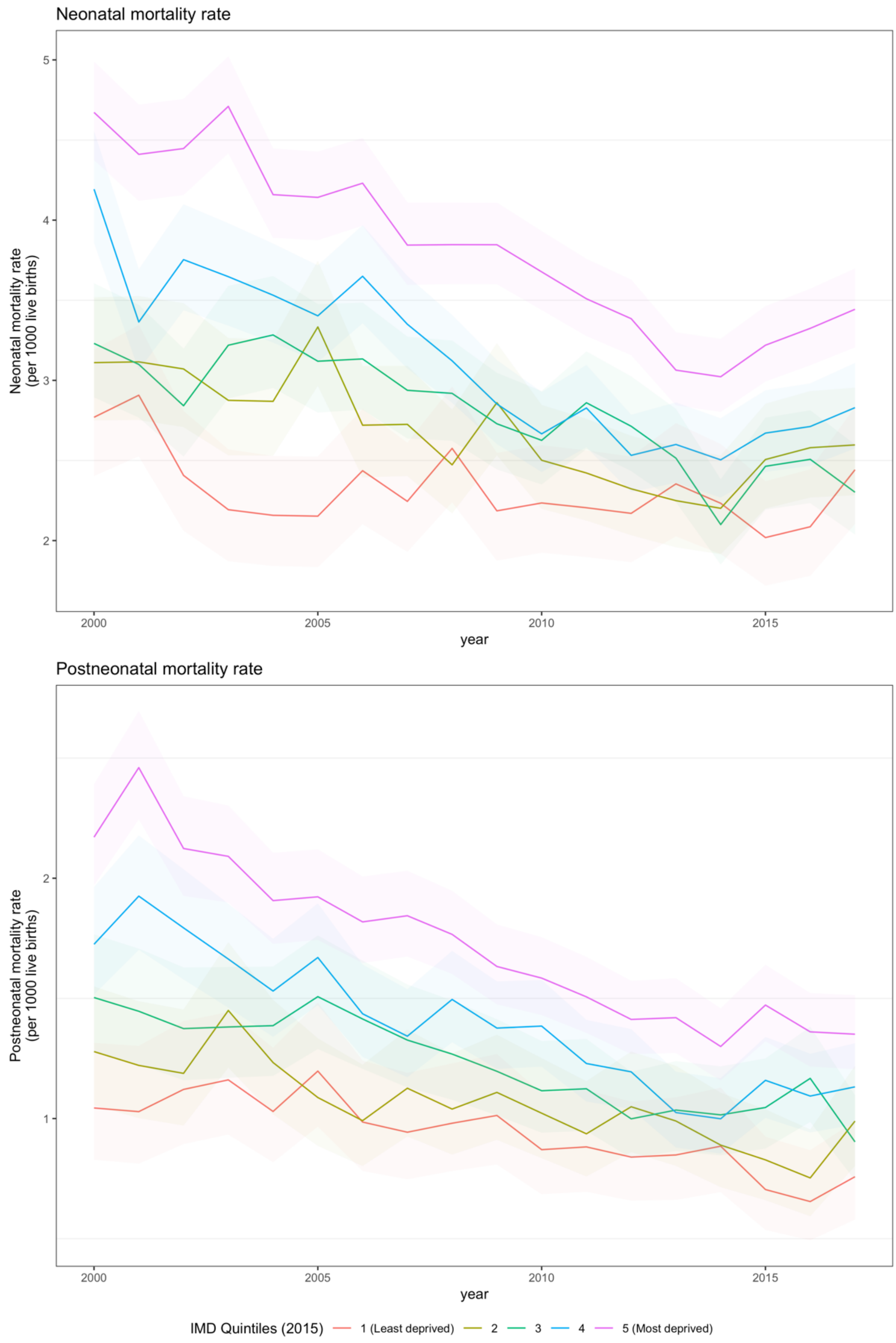

Web appendix 11: Estimates from the main analysis repeated using neonatal mortality and post-neonatal mortality as the outcome, plotted with their confidence intervals.

Estimates for annual change (deaths per 100,000) in neonatal mortality and post-neonatal mortality 2014-2017 relative to previous trend (2000-2013) and their confidence intervals by small area deprivation quintile (most deprived=quintile 5).

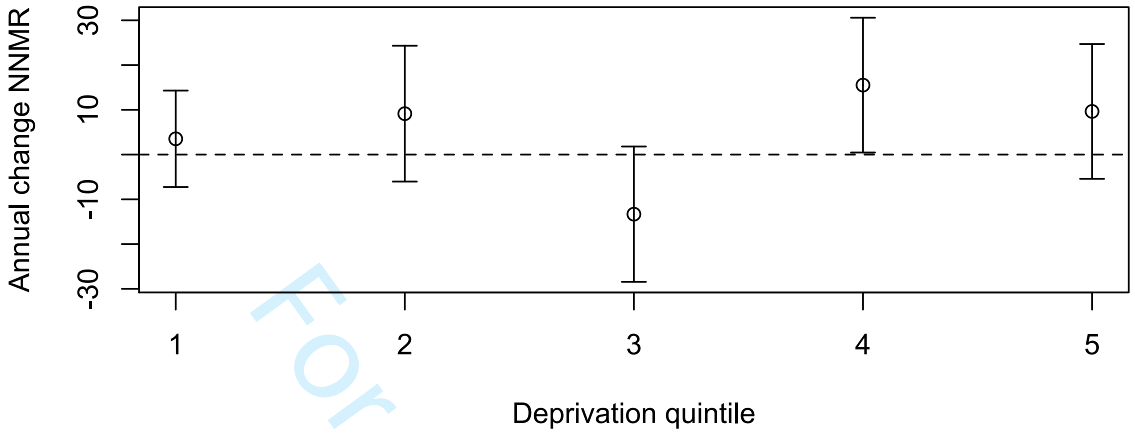

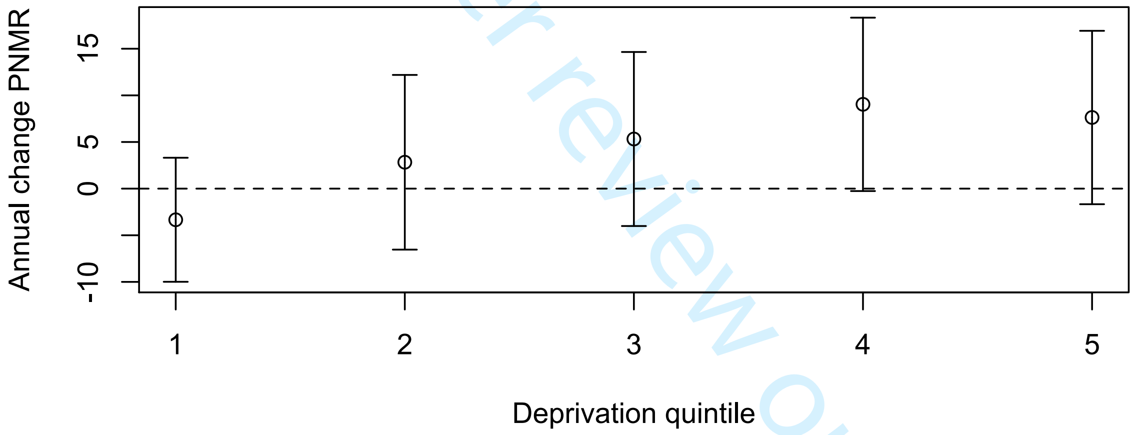

For peer review only

STROBE Statement—checklist of items that should be included in reports of observational studies

	Item No	Recommendation
**Title and abstract**	1	(*a*) Indicate the study's design with a commonly used term in the title or the abstract - Done : Title: **Child poverty and inequalities in infant mortality in England 2000-17: time trend analysis**
		(*b*) Provide in the abstract an informative and balanced summary of what was done and what was found<br>– Done – see ABSTRACT page 2
**Introduction**		
Background/rationale	2	Explain the scientific background and rationale for the investigation being reported. - Done see BACKGROUND page 4
Objectives	3	State specific objectives, including any prespecified hypotheses<br>Done see Abstract and BACKGROUND- "We therefore investigate the extent to which increases in child poverty may have led to increased infant mortality in recent years. Firstly, we investigate whether infant mortality increased more in those parts of the country with the greater numbers of people on a low income or receiving low income related welfare benefits. Secondly, we estimate whether there was a significant change in the trend in infant mortality in those areas and the timing of this change. Thirdly, we exploit regional differences in rates of infant mortality and child poverty to estimate the extent to which those regions with greater increases in child poverty experience greater increases in infant mortality. We use these estimates to calculate the proportion of recent infant mortality increases that are potentially explained by increases in child poverty."<br>Page 5
**Methods**		
Study design	4	Present key elements of study design early in the paper<br>– Done – see METHODS page 5
Setting	5	Describe the setting, locations, and relevant dates, including periods of recruitment, exposure, follow-up, and data collection. Done – see METHODS➔ Setting and data sources page 5
Participants	6	(*a*) *Cohort study*—Give the eligibility criteria, and the sources and methods of selection of participants. Describe methods of follow-up – NA – participants are the entire population<br>*Case-control study*—Give the eligibility criteria, and the sources and methods of case ascertainment and control selection. Give the rationale for the choice of cases and controls- NA<br>*Cross-sectional study*—Give the eligibility criteria, and the sources and methods of selection of participants - NA
		(*b*) *Cohort study*—For matched studies, give matching criteria and number of exposed and unexposed- NA<br>*Case-control study*—For matched studies, give matching criteria and the number of controls per case - NA

For peer review only - http://bmjopen.bmj.com/site/about/guidelines.xhtml

Variables	7	Clearly define all outcomes, exposures, predictors, potential confounders, and effect modifiers. Give diagnostic criteria, if applicable. Done see METHODS➔ Data sources page 5
Data sources/ measurement	8*	For each variable of interest, give sources of data and details of methods of assessment (measurement). Describe comparability of assessment methods if there is more than one group. Done see METHODS➔ Data sources page 5
Bias	9	Describe any efforts to address potential sources of bias- Done see METHODS➔ analysis page 6-7 – fixed effect approach
Study size	10	Explain how the study size was arrived at. NOTE: the study size is the entire population of England, analysed as aggregate data for 324 local authorities and 9 regions.
Quantitative variables	11	Explain how quantitative variables were handled in the analyses. If applicable, describe which groupings were chosen and why. Done see METHODS➔ analysis - page 5 – "Local authorities were grouped in 5 categories (quintiles) based on the income deprivation score of the 2015 Indices of Multiple Deprivation."
Statistical methods	12	(a) Describe all statistical methods, including those used to control for confounding. Done- Done see METHODS➔ analysis- page 6 & 7
		(*b*) Describe any methods used to examine subgroups and interactions Done see METHODS➔ analysis- page 6 &7 – "To do this we used the local authority panel data to estimate a segmented mixed effects regression model for infant mortality including two linear spline terms for time interacted with income deprivation quintile, with a breakpoint indicating the change in trend."
		(*c*) Explain how missing data were addressed- As the measures are based on the whole population this is not relevant. There was no missing data.
		(*d*) *Cohort study*—If applicable, explain how loss to follow-up was addressed - NA<br>*Case-control study*—If applicable, explain how matching of cases and controls was addressed- NA<br>*Cross-sectional study*—If applicable, describe analytical methods taking account of sampling strategy- NA
		(*e*) Describe any sensitivity analyses. Done see robustness tests – page 7. And Web appendix.

Continued on next page

For peer review only - http://bmjopen.bmj.com/site/about/guidelines.xhtml

**Results**		
Participants	13*	(a) Report numbers of individuals at each stage of study—eg numbers potentially eligible, examined for eligibility, confirmed eligible, included in the study, completing follow-up, and analysed- NA
		(b) Give reasons for non-participation at each stage- NA
		(c) Consider use of a flow diagram- NA
Descriptive data	14*	(a) Give characteristics of study participants (eg demographic, clinical, social) and information on exposures and potential confounders –NA
		(b) Indicate number of participants with missing data for each variable of interest- There were no missing data
		(c) *Cohort study*—Summarise follow-up time (eg, average and total amount)- NA
Outcome data	15*	*Cohort study*—Report numbers of outcome events or summary measures over time- NA
		Case-control study—Report numbers in each exposure category, or summary measures of exposure- NA
		Cross-sectional study—Report numbers of outcome events or summary measures- NA
Main results	16	(*a*) Give unadjusted estimates and, if applicable, confounder-adjusted estimates and their precision (eg, 95% confidence interval). Make clear which confounders were adjusted for and why they were included- Done: Unadjusted estimates -see Figure 1 and in web appendix 4, Adjusted estimates in table 1
		(*b*) Report category boundaries when continuous variables were categorized- NA
		(*c*) If relevant, consider translating estimates of relative risk into absolute risk for a meaningful time period- All results are given as both and relative absolute change
Other analyses	17	Report other analyses done—eg analyses of subgroups and interactions, and sensitivity analyses. Done see RESULTS ➔ robustness tests page 8 and web appendix 4.
**Discussion**		
Key results	18	Summarise key results with reference to study objectives. Done see discussion page 8
Limitations	19	Discuss limitations of the study, taking into account sources of potential bias or imprecision. Discuss both direction and magnitude of any potential bias- Done see discussion page 8
Interpretation	20	Give a cautious overall interpretation of results considering objectives, limitations, multiplicity of analyses, results from similar studies, and other relevant evidence- Done see discussion page 8-10
Generalisability	21	Discuss the generalisability (external validity) of the study results. Done see discussion page 10
**Other information**		
Funding	22	Give the source of funding and the role of the funders for the present study and, if applicable, for the original study on which the present article is based- Funding sources given page 11.

*Give information separately for cases and controls in case-control studies and, if applicable, for exposed and unexposed groups in cohort and cross-sectional studies.

Note: An Explanation and Elaboration article discusses each checklist item and gives methodological background and published examples of transparent reporting. The STROBE checklist is best used in conjunction with this article (freely available on the Web sites of PLoS Medicine at http://www.plosmedicine.org/, Annals of Internal Medicine at http://www.annals.org/, and Epidemiology at http://www.epidem.com/). Information on the STROBE Initiative is available at www.strobe-statement.org.

For peer review only - http://bmjopen.bmj.com/site/about/guidelines.xhtml
